# Atomically Dispersed Dual-Metal Sites Showing Unique Reactivity and Dynamism for Electrocatalysis

**DOI:** 10.1007/s40820-023-01080-y

**Published:** 2023-05-01

**Authors:** Jun-Xi Wu, Wen-Xing Chen, Chun-Ting He, Kai Zheng, Lin-Ling Zhuo, Zhen-Hua Zhao, Jie-Peng Zhang

**Affiliations:** 1 MOE Key Laboratory of Bioinorganic and Synthetic Chemistry, School of Chemistry, Sun Yat-Sen University, Guangzhou, 510275 People’s Republic of China; 2 Key Lab of Fluorine and Silicon for Energy Materials and Chemistry of Ministry of Education, College of Chemistry and Chemical Engineering, Jiangxi Normal University, Nanchang, 330022 People’s Republic of China; 3 Energy & Catalysis Center, School of Materials Science and Engineering, Beijing Institute of Technology, Beijing, 100081 People’s Republic of China

**Keywords:** Metal–organic frameworks, Atomically dispersed catalyst, Hydrogen bond, Overall water splitting

## Abstract

The real structure and in situ evolution of catalysts under working conditions are of paramount importance, especially for bifunctional electrocatalysis. Here, we report asymmetric structural evolution and dynamic hydrogen-bonding promotion mechanism of an atomically dispersed electrocatalyst. Pyrolysis of Co/Ni-doped MAF-4/ZIF-8 yielded nitrogen-doped porous carbons functionalized by atomically dispersed Co–Ni dual-metal sites with an unprecedented N8V4 structure, which can serve as an efficient bifunctional electrocatalyst for overall water splitting. More importantly, the electrocatalyst showed remarkable activation behavior due to the in situ oxidation of the carbon substrate to form C–OH groups. Density functional theory calculations suggested that the flexible C–OH groups can form reversible hydrogen bonds with the oxygen evolution reaction intermediates, giving a bridge between elementary reactions to break the conventional scaling relationship.

**Supplementary Information:**

The online version contains supplementary material available at 10.1007/s40820-023-01080-y.

## Highlights

- An atomically dispersed catalyst with unprecedented N8V4 Co-Ni dual-metal sites is synthesized, which shows interesting asymmetric in situ structural evolution and serves as a quasi-bifunctional catalyst for water splitting.
- The flexible C–OH groups generated by in situ oxidation can reversibly turn on/off the hydrogen-bonding interaction with the oxygen evolution reaction intermediates to break the conventional scaling relationship.

**Supplementary Information:**

The online version contains supplementary material available at 10.1007/s40820-023-01080-y.

## Graphical abstract

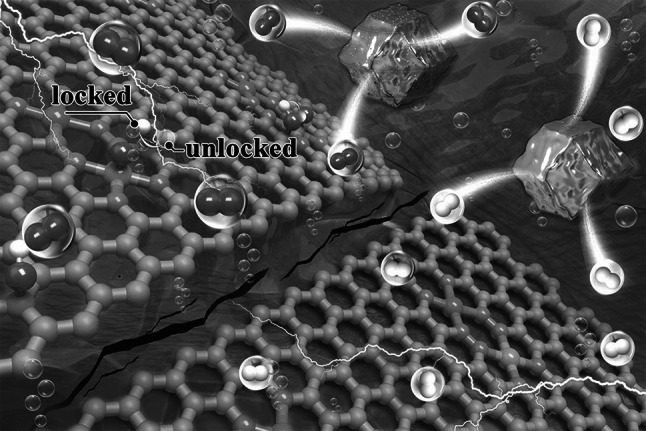

## Introduction

Electrochemical water splitting for hydrogen production requires efficient and stable electrocatalysts that can significantly reduce the overpotentials of both two half reactions, i.e., the hydrogen evolution reaction (HER) on the cathode and the oxygen evolution reaction (OER) on the anode [1–4]. Platinum-based materials and IrO_2_/RuO_2_ are the benchmark electrocatalysts for HER and OER, respectively [5, 6]. In view of the high cost and low earth-abundance of these precious metals, many efforts have been devoted to finding alternative electrocatalysts based on nonprecious metals such as cobalt, nickel, iron, and copper, etc. [7–9]. Transition-metal phosphides/sulfides/selenides/nitrides [10–13] and transition-metal oxides/hydroxides/oxyhydroxides [14–16] are relatively active HER and OER electrocatalysts so far, respectively. While numerous pieces of research have focused on developing of HER/OER bifunctional electrocatalysts for overall water splitting, there are still few successful cases [17, 18], which can be attributed to the different reaction mechanisms of the two half reactions [19–22].

Electrocatalysts usually change their structures during the catalysis/reaction processes. Although structure collapse associated with the inactivation of the catalyst is usually concerned [23–26], some controllable structural changes (such as oxidation, reduction, ion exchange, and exfoliation) that can improve the catalytic performance have drawn extensive interest in recent years [27–30]. Besides in situ structural changes, many catalysts, especially on the particle surfaces, may undergo ex situ changes that are critical for the catalysis performances. For example, the surface of metal phosphides and sulfides would unavoidably be oxidized in air and/or hydrolyzed in water, meaning that such bifunctional electrocatalysts for overall water splitting possess different active structures for OER and HER [31–33].

Showing high atom utilization efficiency, high activity, high selectivity, and simple local structures, atomically dispersed catalysts (ADCs) have demonstrated great potential as heterogeneous catalysts [34–36]. So far, most studies for ADCs still focused on the synthesis, structural characterization, and catalytic mechanisms related to the unique electronic structures and unsaturated coordination environments. These materials are generally assumed to be stable [37], and little attention has been paid to the in situ structural changes during electrocatalytic processes [38–40]. In this work, we synthesized electrocatalysts based on nitrogen-doped porous carbons functionalized by an unprecedented type of atomically dispersed N8V4-CoNi dual-metal sites, which show interesting in situ structural evolution during OER and can be used as efficient quasi-bifunctional electrocatalysts for overall water splitting.

## Experimental and Calculation

### Materials and General Methods

All reagents were commercially available and used without further purification. PXRD patterns were recorded using a Bruker D8 Advance X-ray powder diffractometer (Cu-K*α*) at room temperature. XPS was performed using an ESCA Lab250 X-ray photoelectron spectrometer. All XPS spectra were corrected using the C 1*s* line at 284.8 eV, and curve fitting and background subtraction were accomplished. SEM images were obtained using a HITACHI SU8010 apparatus working at an electron acceleration energy of 5 kV and 10 μA. TEM, HRTEM, and scanned-transmission electron microscopy-EDS element mapping were performed using a JEOL JEM-ARM200F apparatus working at an acceleration voltage of 200 kV. Double Cs-corrected HAADF-STEM was performed using a FEI Titan^3^ Cubed Themis G2 apparatus working at an acceleration voltage of 300 kV. Gas sorption isotherms were measured with a Micromeritics ASAP 2020 M automatic volumetric adsorption apparatus. The temperature was controlled by a liquid-nitrogen bath (77 K). Before the gas sorption experiments, the samples placed in the sample tube were activated under high vacuum at 378 K for 3 h. Raman spectroscopy was conducted using a Renishaw inVia Laser Micro-Raman Spectrometer with a 514.5 nm laser. The *operando* ATR-FTIR measurement was performed using a Nicolet iS50 FT-IR spectrometer equipped with an MCT detector cooled with liquid nitrogen and PIKE EC-IR-II ATR sampling accessory. The spectrum of the as-prepared catalyst at the open-circuit state was used as the baseline of the spectra after applying current. ICP-MS was conducted using a Thermofisher iCAP Qc instrument. XAFS spectra were obtained at 14W1 station in Shanghai Synchrotron Radiation Facility (SSRF) operated at 3.5 GeV with a maximum current of 300 mA. Using Si(111) double-crystal monochromator, the data collection was carried out in the fluorescence excitation mode using a Lytle detector for the samples, and in the transmission mode using an ionization chamber for the references. Co foil, CoO, Co_3_O_4_, Ni foil, and NiO were used as references. All spectra were collected in ambient conditions. All samples were pelletized as disks of 13 mm in diameter and 1 mm in thickness using graphite powder as a binder.

### Synthetic Procedures

#### Synthesis of Co/Ni-doped MAF-4

Similar to synthesis of MAF-4 powders [41], a methanol solution (45 mL) of Zn(NO_3_)_2_·6H_2_O (*z* mmol), Co(NO_3_)_2_·6H_2_O (*c* mmol) and Ni(NO_3_)_2_·6H_2_O (*n* mmol) was poured into a methanol solution (25 mL) of Hmim (1.97 g, 24 mmol) and stirred for 24 h at room temperature. The feeding amounts were summarized in Table S1. The resultant microcrystalline powders were filtered, washed twice with methanol, and dried in air (yield: 60% ~ 65%).

#### Synthesis of AD-Co_x_Ni_1−x_ and NC-Co_0.49_Ni_0.51_

Co/Ni-doped MAF-4 (0.4 g) and NH_4_Cl (1.0 g) were mixed and transferred into a tube furnace in flowing N_2_ atmosphere, heated from room temperature to 900 °C with a rate of 5 °C min^−1^, kept at 900 °C for 3 h, then cooled naturally to room temperature.

### Preparation of Working Electrodes

Unless otherwise stated, the catalyst powder was coated on GCE using Nafion as binder. Rotating disk GCE (0.196 cm^2^, Pine Research Instrumentation) was polished carefully with 1.0, 0.3, and 0.05 μm alumina powder successively, rinsed with ultrapure water, treated by ultrasonication in ethanol and ultrapure water successively, and then dried in air. The catalyst powder (15 mg) was added into a solution (2 mL) containing isopropanol (1.4 mL), water (0.5 mL), and a 5 wt% Dupont Nafion 117 solution (0.1 mL), followed by ultrasonication for 30 min. The suspension (10 μL) was pipetted on the GCE surface (0.38 mg cm^−2^), then dried at ambient temperature before electrochemical measurements. CPE was dipped in a HCl solution (3 mol L^−1^) for 15 min, and then washed with ethanol and water in turn. The coating procedure for CPE was the same as that for GCE, except that the catalyst loading as was 2.0 mg cm^−2^.

### Electrochemical Measurements

All measurements for OER and HER were carried out in a standard three compartment electrochemical glass cell with a CHI 760E (CH Instruments, Inc., Shanghai) electrochemical workstation at room temperature in 1.0 M KOH. GCE was operated at a rotating speed of 1,600 rpm. CV curves were measured at 50 mV s^−1^. LSV curves were measured at a scan rate of 5 mV s^−1^. All potentials were referenced to a saturated calomel electrode (SCE) reference electrode, and a graphite electrode was used as the counter electrode. All potentials were adjusted to compensate for the ohmic potential drop losses (*R*_s_) that arose from the solution resistance and calibrated with respect to the reversible hydrogen electrode (RHE), in which *E*_*vs*.RHE_ = *E*_*vs*.SCE_ + 0.2412 + 0.05916pH − *iR*_s_. The full electrolyzer was measured in a two-electrode cell configuration in 1.0 M KOH solution.

The TOF value is calculated using the following equation:

$$ {\text{TOF }} = \, \left( {J \times A} \right) \, / \, ({4 } \times F \times n) $$

where *J* is the current density at a given overpotential, *A* is the geometric surface area of the electrode, *F* is the Faraday’s constant (96,485.3 C mol^−1^), and *n* is the number of moles of Co and Ni atoms on the working electrode.

### Computational Simulations

In this work, all the geometry optimizations and energy calculations were performed by the spin polarization DFT method through the Cambridge Sequential Total Energy Package (CASTEP) procedure in the Materials Studio 2019 package. The generalized gradient approximation (GGA) with the Perdew-Burke-Ernzerhof (PBE) exchange–correlation functional was adopted for the calculations. The plane wave pseudo-potential method (PWP) with cutoff energy of 571.4 eV for the plane-wave basis set was used. All models were calculated in periodic boxes with a vacuum slab of 15 Å to separate the interaction between periodic images. The simulated unit-cell is hexagonal with 14.63 Å × 14.63 Å × 15.00 Å. The energy, force, stress, atomic displacement, and self-consistent field (SCF) convergence criteria were set to be 1 × 10^−5^ eV atom^−1^, 3 × 10^−2^ eV Å^−1^, 5 × 10^−2^ GPa, 1 × 10^−3^ Å, and 1 × 10^−6^ eV atom^−1^, respectively.

The mechanism for OER in alkaline media and the corresponding Gibbs free energy changes (Δ*G*_*n*_, *n* = 1, 2, 3, and 4) were expressed by (* is an adsorption site on the catalysts):

1 $$ {\text{H}}_{{2}} {\text{O }} + \,^ {*} \, \to {\text{ OH}}^{*} \, + {\text{ e}}^{ - } + {\text{ H}}^{ + }$$

$$ \Delta G_{{1}} = \, \Delta G_{{{\text{OH}}^{*}}} - {\text{e}}U + k_{{\text{B}}} T{\text{ln1}}0 \, \times {\text{ pH}}$$

2 $$ {\text{OH}}^{*} \, \to {\text{ O}}^{*} \, + {\text{ e}}^{ - } + {\text{ H}}^{ + }$$

$$ \Delta G_{{2}} = \, \Delta G_{{{\text{O}}*}} {-} \, \Delta G_{{{\text{OH}}*}} - {\text{e}}U + k_{{\text{B}}} T{\text{ln1}}0 \, \times {\text{ pH}} $$

3 $$ {\text{O}}^{*} \, + {\text{ H}}_{{2}} {\text{O }} \to {\text{ OOH}}^{*} \, + {\text{ e}}^{ - } + {\text{ H}}^{ + } $$

$$ \Delta G_{{3}} = \, \Delta G_{{{\text{OOH}}^{*}}} - \Delta G_{{{\text{O}}^{*}}} - {\text{e}}U + k_{{\text{B}}} T{\text{ln1}}0 \, \times {\text{ pH}} $$

4 $$ {\text{OOH}}^{*} \, \to \, ^{*} \, + {\text{ O}}_{{2}} + {\text{ e}}^{ - } + {\text{ H}}^{ + } $$

$$ \Delta G_{{4}} = { 4}.{\text{92 eV}} - \Delta G_{{{\text{OOH}}^{*}}} - {\text{e}}U + k_{{\text{B}}} T{\text{ln1}}0 \, \times {\text{ pH}} $$

The theoretical overpotential is calculated by:

5 $$ \eta_{{\text{O}}} = {\text{ max}}\left[ {\Delta G_{{1}} , \, \Delta G_{{2}} , \, \Delta G_{{3}} , \, \Delta G_{{4}} } \right]/{\text{e}} - {1}.{\text{23 V}} $$

The free energy of the adsorbed state was calculated as

$$ \Delta G = \, \Delta E_{{{\text{ads}}}} - \Delta E_{{{\text{ZPE}}}} - T\Delta S $$

where Δ*E*_ads_, Δ*E*_ZPE_, and Δ*S* are the reaction energy calculated using DFT, zero-point energy (ZPE) change, and entropy change, respectively. ZPE and Δ*S* were obtained from the vibrational frequencies analysis and the standard table for gas-phase molecules based on previous works, respectively [42, 43].

## Results and Discussion

### Synthesis and Characterization

**Fig. 1:**
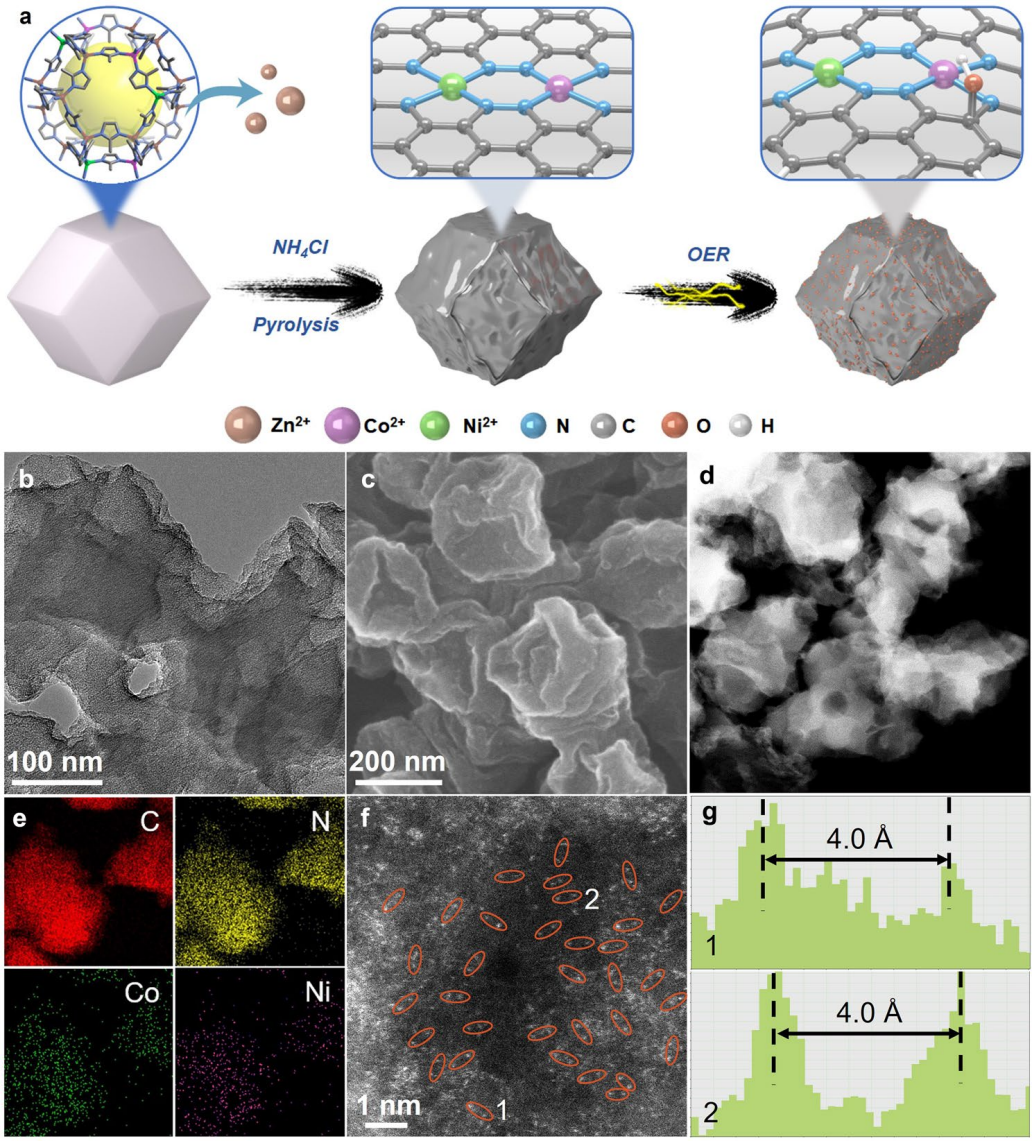
Synthesis and structural characterization. **a** Schematic illustration of the synthetic processes and structures of AD-Co_*x*_Ni_1−*x*_ and O-AD-Co_*x*_Ni_1−*x*_. **b** TEM, **c** SEM, **d** STEM, **e** elemental mapping, **f, g** zoom-in HAADF-STEM and corresponding intensity profiles of AD-Co_0.48_Ni_0.52_

The new ADCs supported by nitrogen-doped porous carbons were synthesized by the typical MOF-pyrolysis method (Figs. 1a and S1–S2). Co/Ni-doped SOD-[Zn(mim)_2_] (MAF-4/ZIF-8 [44, 45], Hmim = 2-methylimidazole) was mixed with NH_4_Cl and then pyrolyzed at 900 °C in N_2_ to obtain the catalyst samples [41]. The Co/Ni contents of the catalysts can be regulated by adjusting the feeding ratio of the Co/Ni/Zn sources for MOF synthesis, and quantitatively determined by inductively coupled plasma-mass spectrometry (ICP-MS). Two broad powder X-ray diffraction (PXRD) peaks at about 26° and 44° associated with the (002) and (101) diffractions of graphitic carbon can be found for all samples (Fig. S3) [46]. When the Co/Ni loading in the sample was lower than 2 wt%, no Co/Ni nanocrystals (NCs) could be observed by PXRD. High-resolution transmission electron microscopy (HRTEM) was further conducted to confirm the absence of metal NCs and/or nanoparticles (Figs. 1b and S4–S9). Six ADCs with total Co/Ni loadings of 1.7–1.8 wt% and different Co:Ni ratios (denoted as AD-Co_1_Ni_0_, AD-Co_0.72_Ni_0.28_, AD-Co_0.62_Ni_0.38_, AD-Co_0.48_Ni_0.52_, AD-Co_0.41_Ni_0.59_, and AD-Co_0_Ni_1_, respectively), as well as an NC catalyst with Co and Ni contents of 2.69 and 2.81 wt%, respectively (denoted as NC-Co_0.49_Ni_0.51_), were used for electrocatalysis studies (Fig. S9). Scanning electron microscopy (SEM) showed bubbled morphologies for all samples (Figs. 1c and S4–S9), which can be attributed to the etching effect of the decomposition products of NH_4_Cl [47]. Besides PXRD, HRTEM, and SEM, more detailed structural characterizations were further performed for AD-Co_1_Ni_0_, AD-Co_0_Ni_1_, AD-Co_0.48_Ni_0.52_ (with the highest electrocatalytic performances), and NC-Co_0.49_Ni_0.51_.

Elemental mapping by energy dispersive X-ray spectrometry (EDS) indicated the homogeneous distribution of C, N, and Co/Ni in AD-Co_1_Ni_0_, AD-Co_0_Ni_1_, and AD-Co_0.48_Ni_0.52_, but aggregated dots of Co/Ni in NC-Co_0.49_Ni_0.51_ (Figs. 1d–e, S10–S12). Double aberration-corrected high-angle annular dark-field scanning transmission electron microscopy (HAADF-STEM) showed isolated bright dots, indicating the presence of atomically dispersed metal sites (Fig. 1f). Interestingly, the distances between many pairs of bright dots were less than 5.0 Å (Figs. 1g and S13), suggesting the existence of dual metal sites such as N8V4 (3.99 Å), N6V4 (2.35 Å), 2 × (N3V2) (2.43 Å), 2 × (N4V2) (5.00 Å), and N7V4 (3.54 Å) (Fig. S14) [46, 48]. It is worth noting that the images of AD-Co_0.48_Ni_0.52_ displayed more such dual bright dots than those of AD-Co_1_Ni_0_ and AD-Co_0_Ni_1_ (Fig. S15), indicating that heterobimetallic doping can facilitate the formation of dual metal sites, being similar to previous works [49]. The N_2_ adsorption of AD-Co_1_Ni_0_, AD-Co_0_Ni_1_, AD-Co_0.48_Ni_0.52_, and NC-Co_0.49_Ni_0.51_ measured at 77 K showed type-I isotherm with a saturated uptake of 23.4 ~ 23.7 mmol g^−1^. The Brunauer–Emmett–Teller (BET) surface areas were calculated as 1305, 1308, 1381, and 1297 m^2^ g^−1^, respectively (Fig. S16). Besides, Raman spectra of AD-Co_1_Ni_0_, AD-Co_0_Ni_1_, AD-Co_0.48_Ni_0.52_, and NC-Co_0.49_Ni_0.51_ showed almost the same *I*_D_/*I*_G_ (peak intensity ratio of the disordered *sp*^3^ carbon at ~ 1350 cm^−1^ and graphite *sp*^2^ carbon at ~ 1590 cm^−1^) values of *ca*. 0.84, implying a similar degree of graphitization (Fig. S17) [50].

To gain more insights into the chemical states of the metal and nitrogen species, X-ray photoelectron spectroscopy (XPS) was conducted. The N 1*s* binding energy of AD-Co_1_Ni_0_, AD-Co_0_Ni_1_, AD-Co_0.48_Ni_0.52_, and NC-Co_0.49_Ni_0.51_ could be deconvoluted into five peaks at ~ 404.5 (oxidized-N), ~ 402.0 (graphitic-N), ~ 400.8 (pyrrolic-N), ~ 399.4 (metal-N), and ~ 398.5 (pyridinic-N) eV, respectively (Fig. S18), being similar to other ADCs [51]. The Co 2*p* binding energy of AD-Co_0.48_Ni_0.52_ was deconvoluted into two pairs of peaks for Co^2+^ (781.6 and 796.2 eV), which were ~ 0.2 eV higher than those of AD-Co_1_Ni_0_ (Fig. S19a). In contrast, the Ni 2*p* binding energy (855.1 and 871.8 eV) of AD-Co_0.48_Ni_0.52_ were ~ 0.2 eV lower than those of AD-Co_0_Ni_1_ (Fig. S19b). These results indicated remarkable electronic interactions between Co and Ni in AD-Co_0.48_Ni_0.52_. The signals of Co^0^ at 778.57 eV and Ni^0^ at 852.8 eV were observed in NC-Co_0.49_Ni_0.51_ instead of AD-Co_1_Ni_0_, AD-Co_0_Ni_1_, and AD-Co_0.48_Ni_0.52_, meaning that the atomically dispersed Co/Ni sites were in the cationic states [52].

**Fig. 2:**
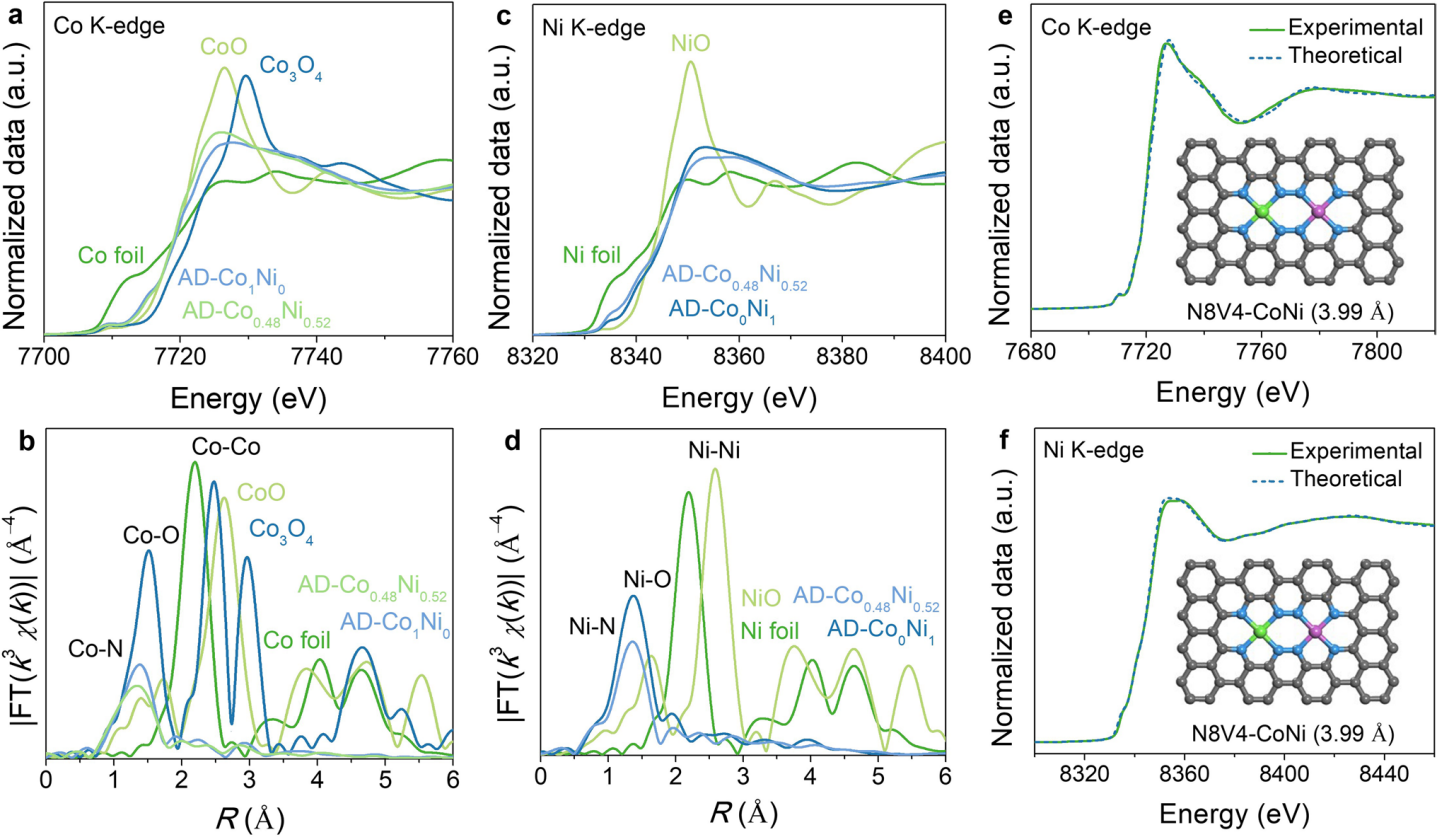
X-ray absorption spectroscopy. **a, b** Co K-edge XANES spectra and corresponding Fourier-transformed EXAFS spectra. **c, d** Ni K-edge XANES spectra and corresponding Fourier-transformed EXAFS spectra. **e** Theoretical Co K-edge XANES spectra of the proposed architecture of N8V4-CoNi. **f** Theoretical Ni K-edge XANES spectra of N8V4-CoNi

The oxidation states of Co/Ni in AD-Co_1_Ni_0_, AD-Co_0_Ni_1_, and AD-Co_0.48_Ni_0.52_ were further studied by X-ray absorption near-edge structure (XANES) spectra. The Co K-edge position of AD-Co_0.48_Ni_0.52_ was *ca.* 4.8 and 0.4 eV higher than those of Co foil and AD-Co_1_Ni_0_, but *ca.* 1.0 and 2.1 eV lower than those of CoO and Co_3_O_4_, respectively (Fig. 2a), while the Ni K-edge position of AD-Co_0.48_Ni_0.52_ was *ca.* 4.6 eV higher than that of Ni foil, but *ca.* 0.5 and 2.1 eV lower than those of AD-Co_0_Ni_1_ and NiO, respectively (Fig. 2c). These results indicated that the valence states of Co and Ni in AD-Co_0.48_Ni_0.52_ are between 0 and + 2, which is typical for ADCs [53, 54]. In addition, the average oxidation states of Co and Ni in AD-Co_0.48_Ni_0.52_ were higher and lower than those in AD-Co_1_Ni_0_ and AD-Co_0_Ni_1_, respectively, being consistent with the XPS results. The coordination environments of AD-Co_1_Ni_0_, AD-Co_0_Ni_1_, and AD-Co_0.48_Ni_0.52_ were studied by Fourier-transformed *k*^3^-weighted extended X-ray absorption fine structure (EXAFS) spectra and wavelet-transformed EXAFS analysis, and compared with those of Co/Ni foil, CoO, Co_3_O_4_, and NiO. A main peak of Co K-edge at *ca.* 1.36 Å and an intensity maximum at *ca.* 3.4 Å^−1^ were observed for both AD-Co_0.48_Ni_0.52_ and AD-Co_1_Ni_0_ (Figs. 2b and S20) [55, 56], which can be assigned to the Co–N scattering path. Similar peak and intensity maximum for the Ni–N scattering path can be observed for AD-Co_0.48_Ni_0.52_ and AD-Co_0_Ni_1_ at *ca.* 1.36 Å and *ca.* 3.2 Å^−1^, respectively (Figs. 2d and S21) [57, 58]. The metal–metal bond peak (*ca.* 2.3 Å) and the intensity maximum (*ca*. 6.8 Å^−1^) for high coordination shells were not detected in AD-Co_1_Ni_0_, AD-Co_0_Ni_1_, and AD-Co_0.48_Ni_0.52_, confirming the atomic dispersion and excluding the possibility of the previously reported N6V4 structure [59, 60], which was synthesized by other precursors.

Further, comparisons of the experimental and theoretical* K*-edge XANES spectra of AD-Co_0.48_Ni_0.52_ showed satisfactory results for the N8V4 model rather than other typical binuclear models, including N6V4, 2 × (N3V2), 2 × (N4V2), and N7V4 (Figs. 2e-f and S22-S23). To the best of our knowledge, although bimetallic catalysts and atomically dispersed dual-metal sites have been reported a lot [34–37, 46, 48, 60], these are the first examples of ADCs with the N8V4-CoNi structures. Quantitative fitting of the EXAFS curve of AD-Co_0.48_Ni_0.52_ gave Co–N bond lengths of 1.93 and 1.97 Å and Ni–N bond lengths of 1.94 and 1.96 Å, respectively (Figs. S24–S25 and Table S2).

### Activation Behaviors for OER

**Fig. 3:**
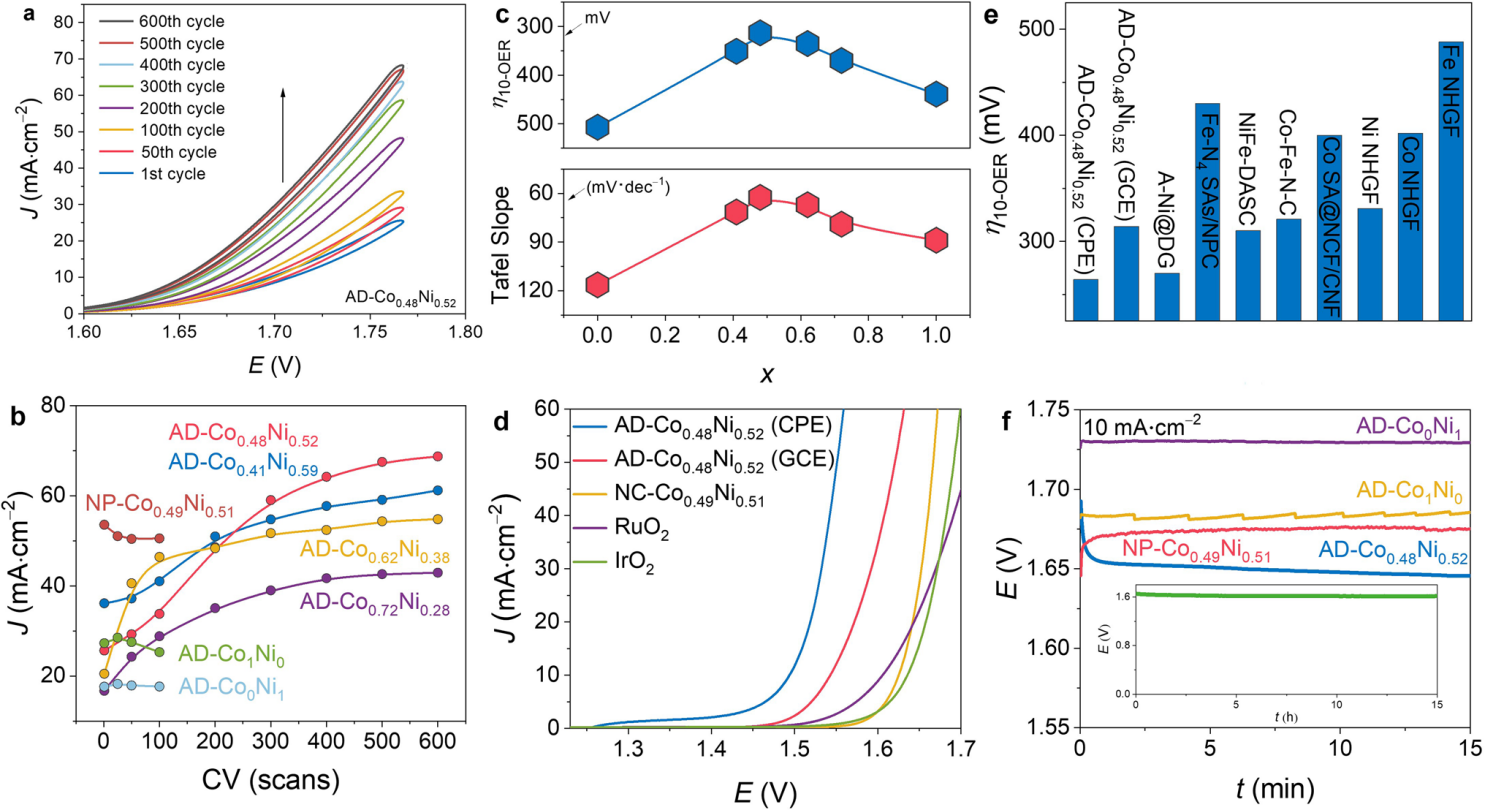
OER behaviors. **a** CV curves of AD-Co_0.48_Ni_0.52_. **b** Variation of the current density (at 1.77 V) with the number of CV scans. **c** Overpotentials and Tafel slopes of AD-Co_*x*_Ni_1−*x*_. **d** LSV curves. **e** Comparison of overpotential of state-of-the-art atomically dispersed electrocatalysts. **f** Chronopotentiometry curves at 10 mA cm^−2^

The electrocatalytic activities of AD-Co_*x*_Ni_1−*x*_ and a series of reference materials (including the commercial IrO_2_, RuO_2_, Pt/C-20%, and NC-Co_0.49_Ni_0.51_) coated on glass carbon electrode (GCE) for OER and HER were investigated in O_2_-saturated and H_2_-saturated 1.0 M KOH, respectively. The electrocatalysts were first stabilized/activated with multiple cyclic voltammetry (CV) scans. In the OER setting, the CV curves of AD-Co_1_Ni_0_, AD-Co_0_Ni_1_, and NC-Co_0.49_Ni_0.51_ can stabilize before 50 cycles like conventional catalysts, but those of AD-Co_*x*_Ni_1−*x*_ (0 < *x* < 1) showed obvious activation behaviors until approximate 500 cycles (Figs. 3a and S26), implying in situ structural evolution of the electrocatalysts. When *x* was close to 0.5 or the Co:Ni ratio was close to 1, the activation process was the most obvious (Fig. 3b), indicating that the synergistic effect of the heterobimetallic site was the key to the activation. After CV stabilization/activation, the OER performances were investigated using linear sweep voltammetry (LSV). With the increase of *x*, the OER overpotential at 10 mA cm^−2^ (*η*_10-OER_) of activated AD-Co_*x*_Ni_1−*x*_ first decreased from 507 mV (*x* = 0) to 313 mV (*x* = 0.48), and then increased to 439 mV (*x* = 1) (Fig. 3c), meaning that the highest activity appeared near *x* = 0.5 again.

The *η*_10-OER_ value of activated AD-Co_0.48_Ni_0.52_ was much lower than those of not only NC-Co_0.49_Ni_0.51_ (395 mV) but also the commercial benchmark RuO_2_ (376 mV) and IrO_2_ (406 mV) (Fig. 3d-e). The Tafel slope (62.5 mV dec^−1^) of activated AD-Co_0.48_Ni_0.52_ was also the lowest among the tested electrocatalysts (Figs. 3c and S27). The activation behavior of AD-Co_0.48_Ni_0.52_ was further demonstrated by chronopotentiometric measurements, in which the potential dramatically decreased over the first 1 h and then maintained nearly constant for 15 h (Fig. 3f). Under the same condition, the chronopotentiometric curves of AD-Co_1_Ni_0_, AD-Co_0_Ni_1_, and NC-Co_0.49_Ni_0.51_ were completely stabilized within 1 min. Raman spectra (Fig. S28) showed that the *I*_D_/*I*_G_ ratio for AD-Co_0.48_Ni_0.52_ after OER (0.943) is higher than that of the initial catalyst (0.849), indicating that the OER/activation process can distort the carbon matrix of the catalysts. Nevertheless, PXRD, SEM, and HRTEM showed that the catalysts were not destroyed to form any identifiable species, such as NCs or nanoparticles of metal or metal oxide/hydroxide (Fig. S29).

**Fig. 4:**
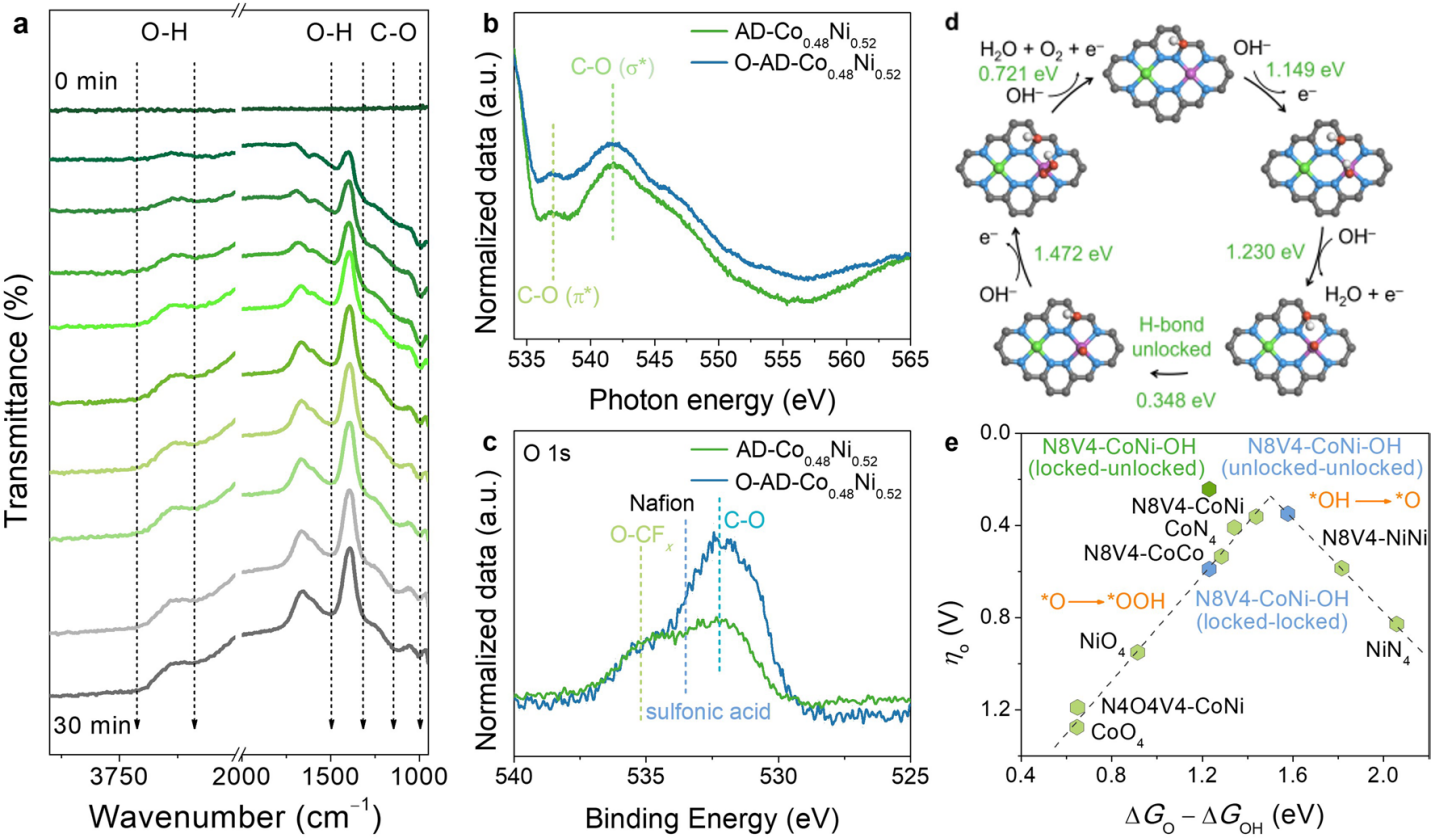
OER and in situ oxidation mechanisms. **a** *Operando* ATR-FTIR of AD-Co_0.48_Ni_0.52_. **b, c** O *K*-edge NEXAFS spectra and high resolution O 1*s* XPS spectra of AD-Co_0.48_Ni_0.52_ and O-AD-Co_0.48_Ni_0.52_. **d** Proposed OER mechanism for O-AD-Co_0.48_Ni_0.52_ (N8V4-CoNi-OH). **e** Volcano relationship between *η*_O_ and Δ*G*_O_ − Δ*G*_OH_

The OER activation mechanism of AD-Co_0.48_Ni_0.52_ was further investigated by *operando* electrochemical attenuated total reflection Fourier transform infrared spectroscopy (ATR-FTIR). After applying the current, absorption peaks at 1000–1300 cm^−1^ (stretching vibration of C–O), 1490 cm^−1^ (bending vibration of O–H), and 3500–3700 cm^−1^ (stretching vibration of O–H) appeared, and the intensities of these peaks gradually increased until *ca*. 30 min (Fig. 4a), whose trend was similar with the chronopotentiometric curve (Fig. S30). No peak of C = O (*ca.* 1710 cm^−1^) was observed in the whole electrochemical process, indicating the formation of C–OH instead of C=O groups. The O *K*-edge near-edge X-ray absorption fine structure (NEXAFS) spectra and the O 1*s* XPS spectra also confirmed the generation of C–OH groups in activated/oxidized AD-Co_0.48_Ni_0.52_ (denoted as O-AD-Co_0.48_Ni_0.52_) (Fig. 4b–c and Table S3) [61].

The OER mechanism was also studied by spin-polarized density functional theory (DFT), using N8V4-CoNi, CoN_4_, CoO_4_, NiN_4_, NiO_4_, N8V4-CoCo, N8V4-NiNi, and N4O4V4-CoNi metal sites inlaid in single-layer graphene as models (Fig. S31). The formation of heteronuclear Co–Ni dual metal sites tailored the d-band center for N8V4-CoNi upward to the Fermi level (Fig. S32), which is beneficial to the adsorption of OER intermediates. Interestingly, the graphene matrix of the N8V4-CoNi model severely bent after geometry optimization (Figs. S33a–c), while others just slightly bent and even kept planar (Figs. S33d–i). The bending of the graphene matrix can weaken *π* conjugation and increase *sp*^3^ hybridization to facilitate oxidative addition (Table S4). According to the experimental results and calculated energies (Table S5), the preferred oxidized position on N8V4-CoNi was found to be the carbon nearby the Co atom, and this model, namely N8V4-CoNi-OH, was used to study the OER mechanism of AD-CoNi.

OER consists of four elementary reactions (Fig. 4d), whose energy barriers depend on the binding abilities of the catalyst toward the reaction intermediates OH, O, and OOH, and the rate-determining step (RDS) possesses the highest energy barrier. According to the common scaling relationship between the binding energies for OH and OOH (Fig. S34), the change of binding energies from OH to O, i.e., Δ*G*_O_ − Δ*G*_OH_, is commonly used as the typical descriptor for the catalytic activity [62]. By plotting theoretical overpotential (*η*_O_) as a function of Δ*G*_O_ − Δ*G*_OH_, a volcano relationship was observed as expected (Fig. 4e). The left and right legs of the volcano indicated that the RDSs are *O → *OOH and *OH → *O due to the relatively strong and weak binding of O, respectively.

In the obtained plot, all the models consisting of O ligands located on the left leg showed higher *η*_O_ than those consisting of N ligands, which ruled out the possibility of these O-ligand models, being similar to the literature results [63]. As expected, *η*_O_ of CoN_4_ (410 mV) and N8V4-NiNi (585 mV) were lower than that of NiN_4_ (828 mV) because O adsorption on Ni is generally too weak [63–65]. Also, *η*_O_ of CoN_4_ (410 mV) was lower than that of N8V4-CoCo (533 mV) because the Co–O binding is generally a little too strong [64, 65]. More importantly, *η*_O_ of N8V4-CoNi (362 mV) was significantly lower than those of all mononuclear and homo-binuclear sites, which can be attributed to the synergetic electronic interactions of the Co–Ni dual-metal sites.

The C–OH group in N8V4-CoNi-OH can form a hydrogen bond (H-bond) with the OER intermediates (Fig. 4d). Considering the rotational flexibility of C-O–H, it can serve as an H-bond donor to lock *O for one elementary reaction and unlock for the other reaction. The switch from the locked state to the unlocked state possesses a negligibly low energy barrier of 0.348 eV, but this additional reaction step changed the energy barriers of *OH → *O and *O → *OOH to 1.230 and 1.472 eV, respectively. For comparison, assuming a rigid C–OH group, the energy barriers of *OH → *O and *O → *OOH were 1.230/1.578 and 1.820/1.472 eV for the locked/unlocked models, respectively. In other words, the energy barrier of the RDS was reduced from 1.820/1.578 eV of the rigid locked/unlocked models to 1.472 eV of the flexible model. The calculation results gave *η*_O_ of 590, 348, and 242 mV for the rigid-locked, rigid-unlocked, and flexible models, respectively. It is worth noting that the value of the flexible model is significantly higher than the volcano plot. Apparently, the on–off switching of the H-bond between the flexible C–OH group and *O can break the scaling relationship in this system (Fig. S35). After oxidation, the d-band center of Co site decreased significantly, which weakened the adsorption of OER intermediates (Fig. S36). However, it was not difficult to notice that the regulation of this electronic structure has little difference in the reaction energy barriers. And the RDS was only reduced from 1.592 to 1.578 eV, which is consistent with the typical scaling relationship. When H-bond is formed, it can break this scaling relationship and significantly regulate the energy barrier of each step.

### HER and Overall Water Splitting

**Fig. 5:**
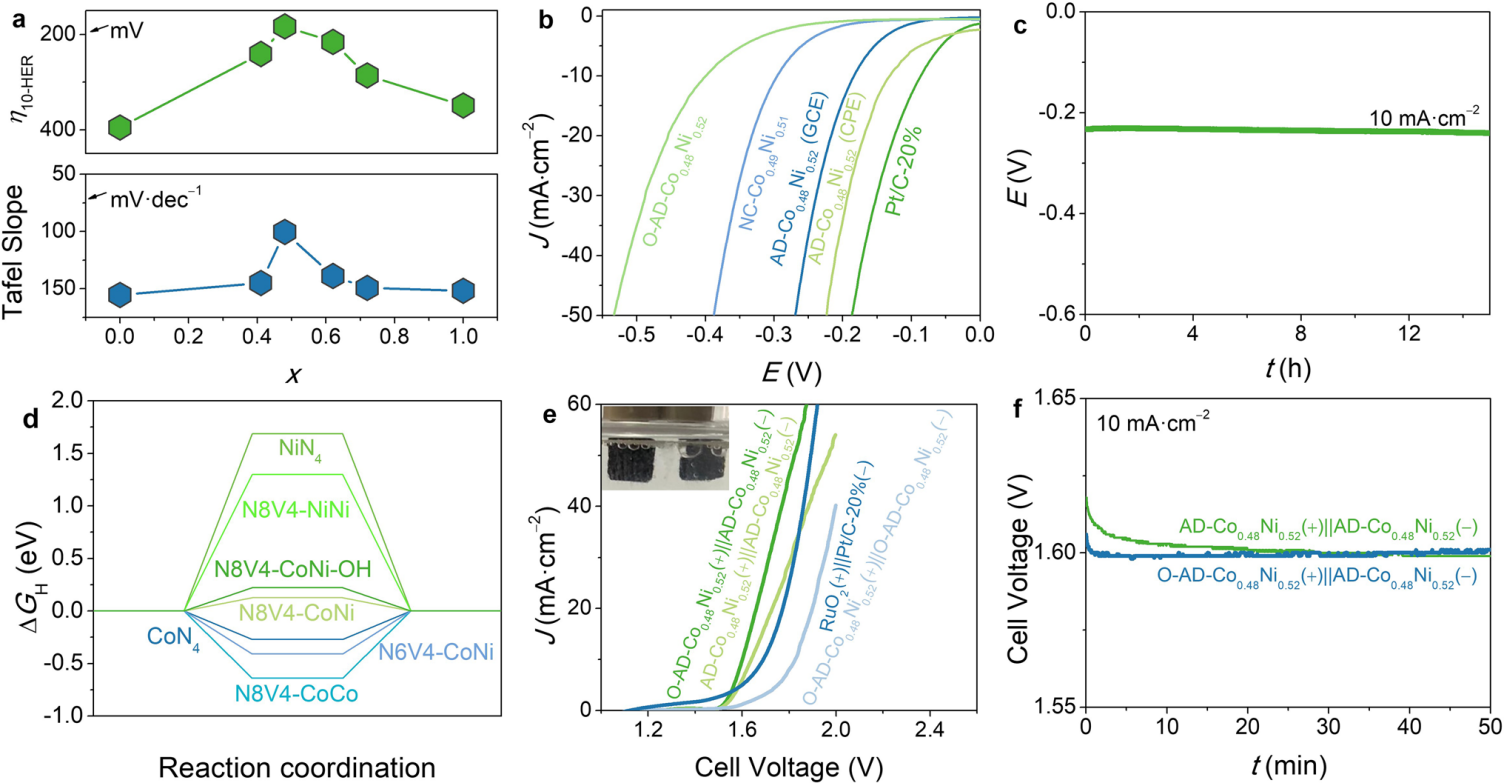
HER and overall water splitting activities. **a** HER overpotentials and Tafel slopes of AD-Co_*x*_Ni_1−*x*_. **b** HER LSV curves. **c** HER chronopotentiometry curve of AD-Co_0.48_Ni_0.52_ at a 10 mA cm^–2^. **d** Calculated Δ*G*_H_ diagram. **e** LSV curves of overall water splitting (inset: photograph of O-AD-Co_0.48_Ni_0.52_( +)‖AD-Co_0.48_Ni_0.52_( −) operating at 10 mA cm^–2^). **f** The chronopotentiometry curves of AD-Co_0.48_Ni_0.52_( +)‖AD-Co_0.48_Ni_0.52_( −) and O-AD-Co_0.48_Ni_0.52_( +)‖AD-Co_0.48_Ni_0.52_( −) at 10 mA cm^–2^ for overall water splitting

For HER, CV showed no activation behavior for all catalysts (Fig. S37). LSV showed that the HER overpotential at 10 mA cm^−2^ (*η*_10-HER_, 183 mV) and Tafel slope (100.3 mV dec^−1^) of AD-Co_0.48_Ni_0.52_ were lower than those of not only other AD-Co_*x*_Ni_1−*x*_ catalysts but also NC-Co_0.49_Ni_0.51_ and O-AD-Co_0.48_Ni_0.52_ (Figs. 5a-c and S38), indicating the synergetic electronic interaction in N8V4-CoNi is also useful for HER, while the C–OH group is adverse to HER.

The free energy (Δ*G*_H_) for adsorption of the H atom on the catalyst (*H) was calculated to explain the different HER activities of the CoN_4_, NiN_4_, N8V4-CoCo, N8V4-NiNi, N8V4-CoNi, and N8V4-CoNi-OH models [66]. The reported N6V4-CoNi model was also calculated for comparison. As shown in Fig. 5d, CoN_4_ exhibited strong H adsorption with Δ*G*_H_ =  − 0.271 eV, and N8V4-CoCo showed much stronger affinity with Δ*G*_H_ =  − 0.637 eV. On the contrary, the H adsorption on NiN_4_ and N8V4-NiNi were extremely weak with large positive Δ*G*_H_ of 1.688 and 1.298 eV, respectively. Due to the synergy between the strong and weak adsorption metal sites, N8V4-CoNi gave appropriate H adsorption with Δ*G*_H_ = 0.126 eV, being consistent with the experimental results. The H adsorption was still so strong on N6V4-CoNi (Δ*G*_H_ =  − 0.407 eV), which may be caused by much stronger electron-interaction on the metals that are too close together (Fig. S39) [48]. Finally, N8V4-CoNi-OH (Δ*G*_H_ = 0.222 eV) displayed a weaker H adsorption than N8V4-CoNi, which can be attributed to the electron withdrawing effect of the C–OH group that reduced the electron density on the metal center [67]. Furthermore, considering the importance of the water dissociation process for HER, the adsorption energy and dissociation energy of H_2_O and the adsorption energy of OH were also calculated. As shown in Fig. S40a, N8V4-CoNi and N6V4-CoNi exhibited lower adsorption energy of H_2_O compared with those other four models, indicating that the adsorption of water molecules on these two heteronuclear diatomic structures was more suitable. The H_2_O dissociation energy (Δ*G*_w_) of N8V4-CoNi was also lower than those of all mononuclear and homo-binuclear sites. As expected, Δ*G*_w_ of N6V4-CoNi is the lowest (Fig. S40b), but its adsorption for OH is far stronger than other models (Fig. S40c). In a word, N8V4-CoNi is the most favorable model for HER.

The turnover frequency (TOF) of AD-Co_0.48_Ni_0.52_ (GCE) at an overpotential of 300 mV for OER and 200 mV for HER reached 0.49 and 2.02 s^−1^, respectively, being similar to those of the state-of-the-art atomically dispersed electrocatalysts (Fig. S41). When AD-Co_0.48_Ni_0.52_ was coated on the carbon paper electrode (CPE), the OER activation process also can be observed during chronopotentiometry. After activation, *η*_10-OER_ and *η*_10-HER_ reached 264 mV and 132 mV, respectively (Figs. 3d, 5b and S27, S38, S42), which were much lower than the values of most ADCs and even comparable with state-of-the-art bifunctional catalysts (Table S6) [68]. Meanwhile, Co and Ni cannot be detected by inductively coupled plasma-atomic emission spectrometer (ICP-AES, Co for 0.01 mg L^–1^ and Ni for 0.02 mg L^–1^) in the electrolyte after long-term electrocatalysis testing, indicating negligible dissolution of Co or Ni during electrocatalysis. AD-Co_0.48_Ni_0.52_ can be used as bifunctional OER/HER catalysts to assemble a water electrolyzer showing higher performance than RuO_2_( +)‖Pt/C-20%(–) (Fig. 5e). Chronopotentiometry of the water electrolyzer showed activation behavior similar with OER (Figs. 5f and S43). The stabilized cell voltage of 1.60 V was lower than most reported non-noble-metal electrocatalysts (Table S7) [19]. Moreover, after 15 h of chronopotentiometry, the cell voltage just increased by 1% from 1.60 to 1.61 V (Fig. S43). When O-AD-Co_0.48_Ni_0.52_ and AD-Co_0.48_Ni_0.52_ were used as the OER and the HER catalysts, respectively, the activation phenomenon disappeared (Fig. 5e–f), confirming that the bifunctional catalyst actually possessed different structures at the OER (activated/oxidized AD-Co_0.48_Ni_0.52_ or N8V4–CoNi–OH) and HER (AD–Co_0.48_Ni_0.52_ or N8V4–CoNi) working conditions. Nevertheless, AD-Co_0.48_Ni_0.52_ can be considered as a quasi-bifunctional electrocatalyst in this system because the two electrodes were prepared with the same material/method.

## Conclusions

In summary, we synthesized atomically dispersed Co–Ni dual-metal sites embedded in nitrogen-doped carbon as a quasi-bifunctional electrocatalyst for overall water splitting. The unprecedented N8V4-CoNi dual-metal sites not only exhibit exceptionally high OER, HER, and water splitting activities, but also promote the in situ oxidation of the carbon matrix to form the C–OH group to further improve the catalytic performance. The flexible C–OH groups can switch between H-bond donor/acceptor states to interact with the OER intermediates, which serve as an adaptive bridge between two elementary reactions of OER to boost the catalytic activity beyond the common volcano-plot limits governed by the scaling relationship. We believe that catalyst flexibility such as the lock*/*unlock pathway can play similar roles for other types of catalytic sites to accelerate the whole reaction. These results provide a new understanding of the structure evolution of ADCs under working conditions, especially for multifunctional electrocatalysts.

### Supplementary Information

Below is the link to the electronic supplementary material.

Supplementary file1 (PDF 9325 KB)
