# Supplementary material for: Atomically Dispersed Dual-Metal Sites Showing Unique Reactivity and Dynamism for Electrocatalysis
Source: Nanomicro Lett. 2023 May 1;15:120. doi: 10.1007/s40820-023-01080-y (PMC10151301; doi:10.1007/s40820-023-01080-y)
Supplement: Supplementary file 1 — Supplementary file1 (PDF 9325 KB) [file 40820_2023_1080_MOESM1_ESM.pdf]

Supporting Information for

# Atomically Dispersed Dual-Metal Sites Showing Unique Reactivity and Dynamism for Electrocatalysis

Jun-Xi Wu[1,#], Wen-Xing Chen[3,#], Chun-Ting He[1,2,*], Kai Zheng[1], Lin-Ling Zhuo[1], Zhen-Hua Zhao[1], and Jie-Peng Zhang[1,*]

[1]MOE Key Laboratory of Bioinorganic and Synthetic Chemistry, School of Chemistry, Sun Yat-Sen University, Guangzhou 510275, P. R. China

[2]Key Lab of Fluorine and Silicon for Energy Materials and Chemistry of Ministry of Education, College of Chemistry and Chemical Engineering, Jiangxi Normal University, Nanchang 330022, P. R. China

[3]Energy & Catalysis Center, School of Materials Science and Engineering, Beijing Institute of Technology, Beijing 100081, P. R. China

[#]Jun-Xi Wu and Wen-Xing Chen contributed equally to this work.

*Corresponding author. E-mail: hct@jxnu.edu.cn (C.-T. He),

zhangjp7@mail.sysu.edu.cn (J.-P. Zhang)

### S1 Text XAFS Data Processing

The acquired EXAFS data were extracted and processed according to the standard procedures using the ATHENA module implemented in the IFEFFIT software packages. The $k^3$-weighted EXAFS spectra were obtained by subtracting the post-edge background from the overall absorption and then normalized with respect to the edge-jump step. Subsequently, $k^3$-weighted $\chi(k)$ data of Co K-edge and Ni K-edge were Fourier transformed to real (R) space using a Hanning window (d$k$=1.0 Å$^{-1}$) to separate the EXAFS contributions from different coordination shells. To obtain the quantitative structural parameters around central atoms, least-squares curve parameter fitting was performed using the ARTEMIS module of IFEFFIT software packages [1].
The following EXAFS equation was used:

$$\chi(k)=\sum_j \frac{N_j S_o^2 F_j(k)}{k R_j^2}\exp[-2k^2\sigma_j^2]\exp[\frac{-2R_j}{\lambda(k)}]\sin[2kR_j+\phi_j(k)]$$

Where $S_0^2$ is the amplitude reduction factor, $F_j(k)$ is the effective curved-wave backscattering amplitude, $N_j$ is the number of neighbors in the $j^{th}$ atomic shell, $R_j$ is the distance between the X-ray absorbing central atom and the atoms in the $j^{th}$ atomic shell (backscatterer), $\lambda$ is the mean free path in Å, $\phi_j(k)$ is the phase shift (including the phase shift for each shell and the total central atom phase shift), $\sigma_j$ is the Debye-Waller parameter of the $j^{th}$ atomic shell (variation of distances around the average $R_j$). The functions $F_j(k)$, $\lambda$ and $\phi_j(k)$ were calculated with the ab initio code FEFF8.2.

## S2 Supplementary Figures and Tables

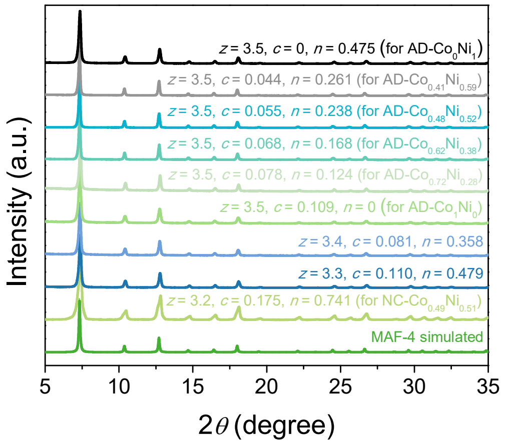


**Fig. S1.** PXRD patterns of Co/Ni-doped MAF-4.

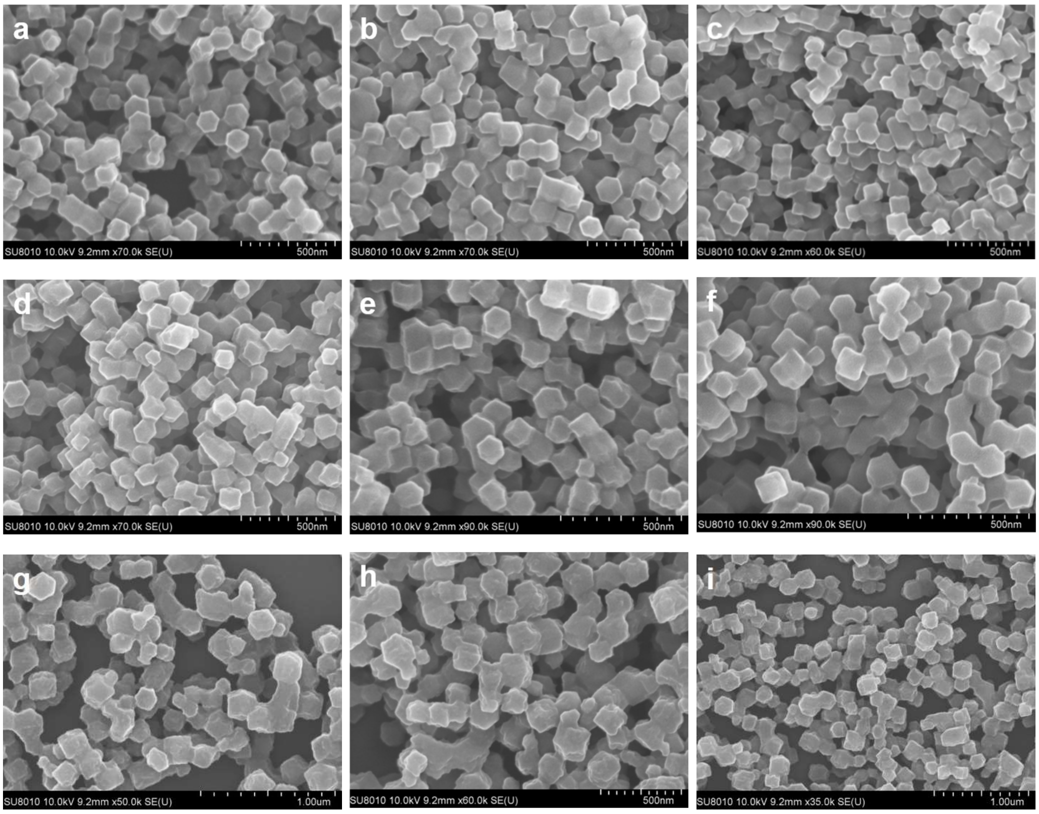


**Fig. S2.** SEM images of Co/Ni-doped MAF-4. (a) $z = 3.5$, $c = 0.109$, $n = 0$ (for AD-$Co_1Ni_0$), (b) $z = 3.5$, $c = 0.078$, $n = 0.124$ (for AD-$Co_{0.72}Ni_{0.28}$), (c) $z = 3.5$, $c = 0.068$, $n = 0.168$ (for AD-$Co_{0.62}Ni_{0.38}$), (d) $z = 3.5$, $c = 0.055$, $n = 0.238$ (for AD-$Co_{0.48}Ni_{0.52}$), (e) $z = 3.5$, $c = 0.044$, $n = 0.261$ (for AD-$Co_{0.41}Ni_{0.59}$), (f) $z = 3.5$, $c = 0$, $n = 0.475$ (for AD-$Co_0Ni_1$), (g) $z = 3.4$, $c = 0.081$, $n = 0.358$, (h) $z = 3.3$, $c = 0.110$, $n = 0.479$, (i) $z = 3.2$, $c = 0.175$, $n = 0.741$ (for NC-$Co_{0.49}Ni_{0.51}$).

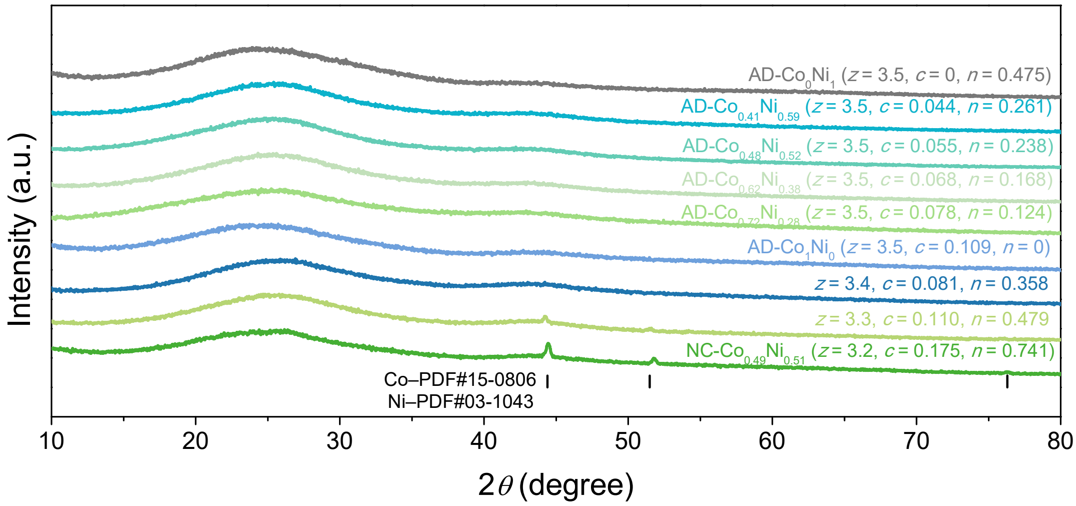


**Fig. S3.** PXRD patterns of the pyrolysis products of Co/Ni-doped MAF-4.

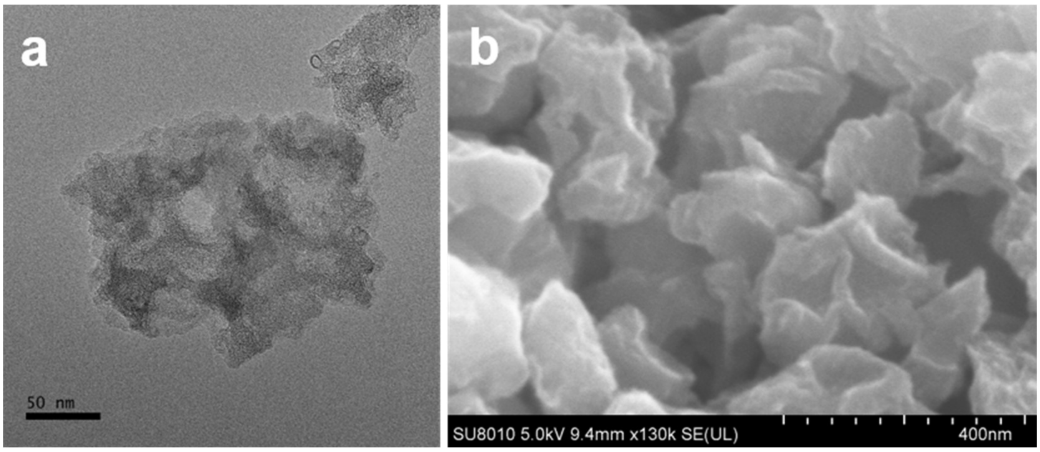


**Fig. S4.** (a) TEM and (b) SEM images of AD-$Co_1Ni_0$.

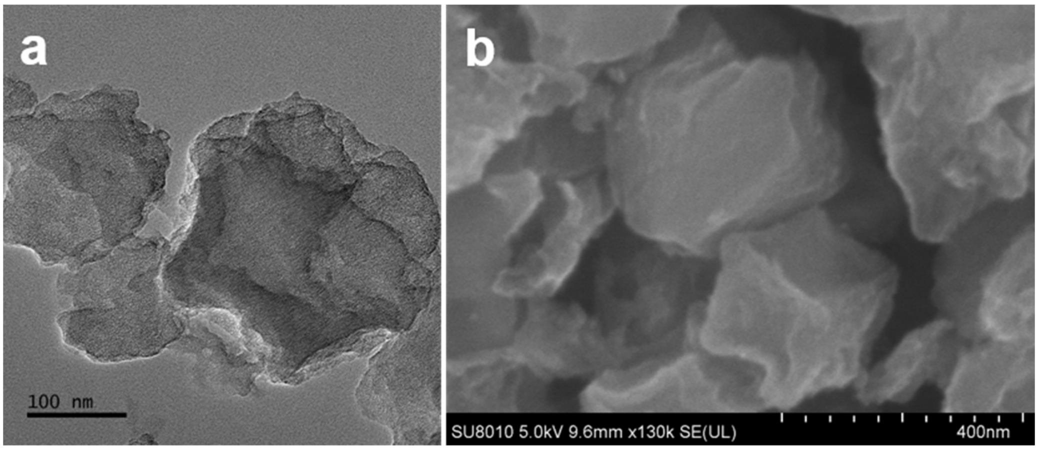


**Fig. S5.** (a) TEM and (b) SEM images of AD-$Co_{0.72}Ni_{0.28}$.

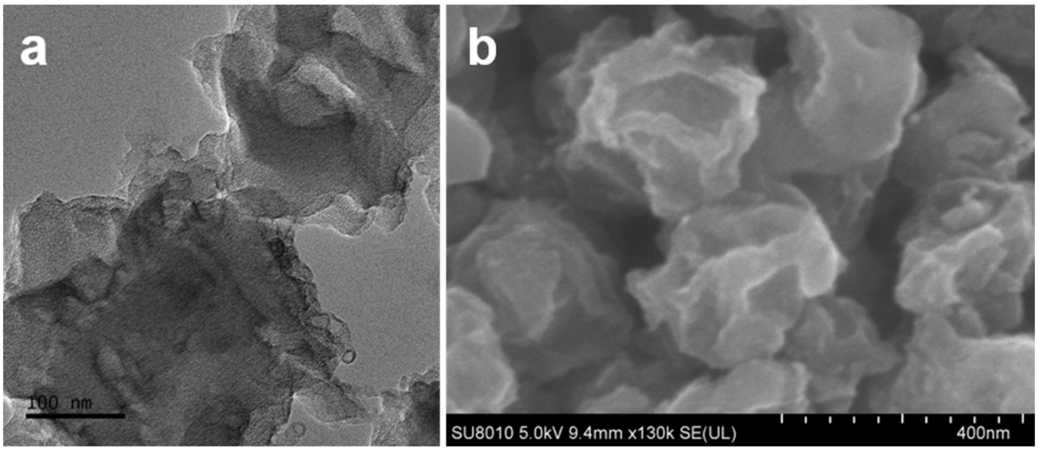

**Fig. S6.** (a) TEM and (b) SEM images of AD-$Co_{0.62}Ni_{0.38}$.

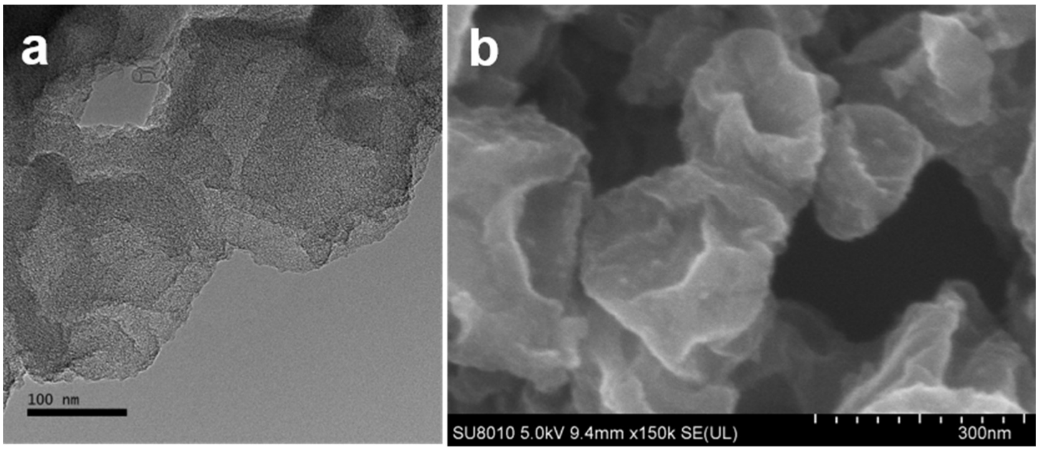

**Fig. S7.** (a) TEM and (b) SEM images of AD-$Co_{0.41}Ni_{0.59}$.

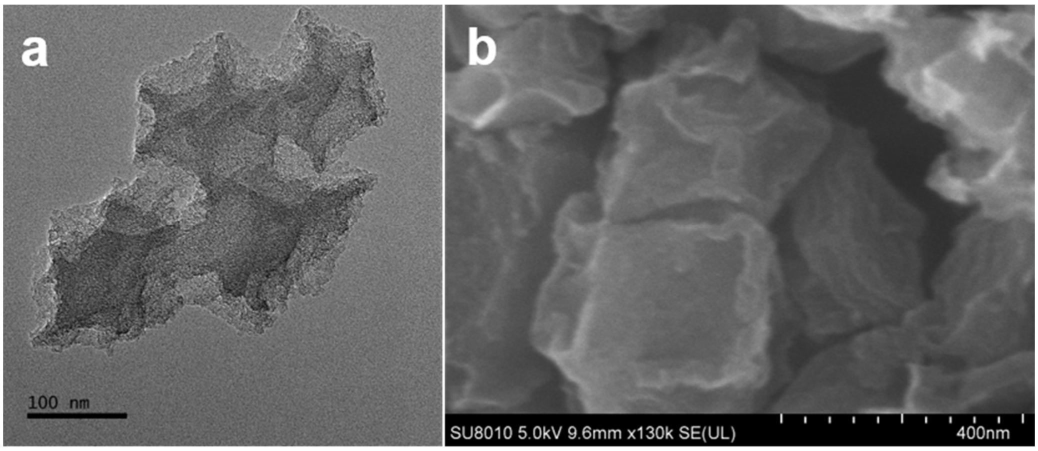

**Fig. S8.** (a) TEM and (b) SEM images of AD-$Co_0Ni_1$.

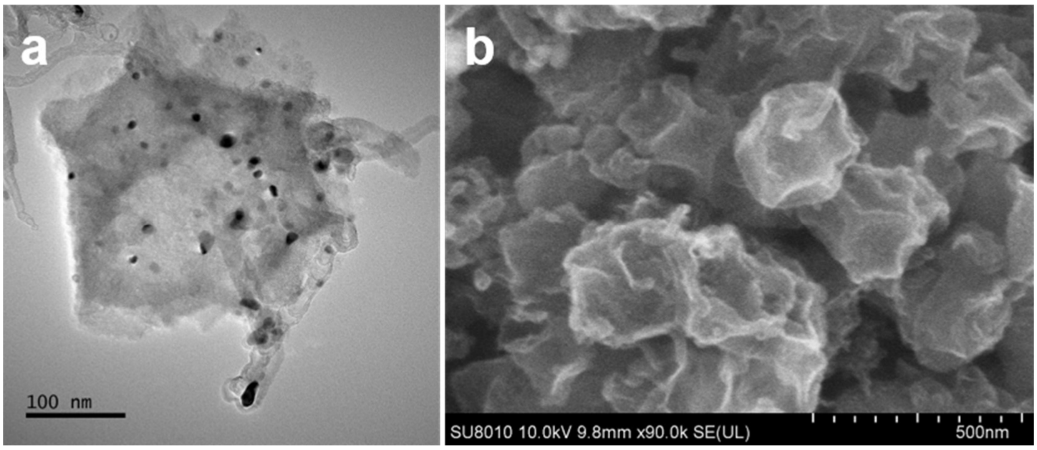

**Fig. S9.** (a) TEM and (b) SEM images of NC-$Co_{0.49}Ni_{0.51}$.

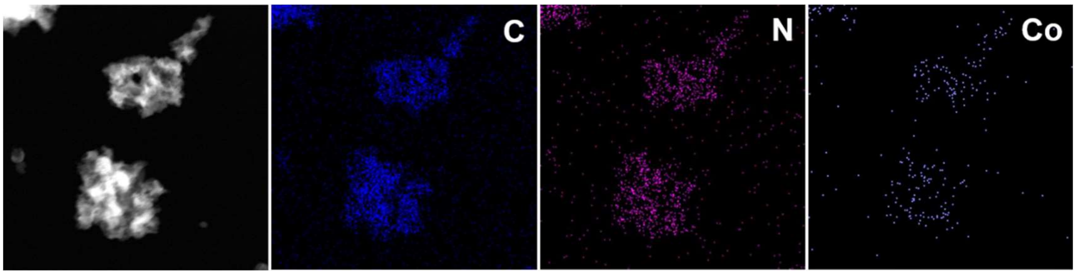

**Fig. S10.** HAADF-STEM and EDS of AD-$Co_1Ni_0$.

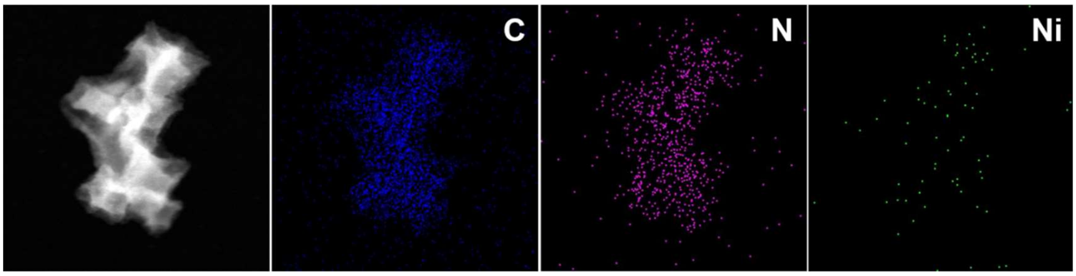

**Fig. S11.** HAADF-STEM and EDS of AD-$Co_0Ni_1$.

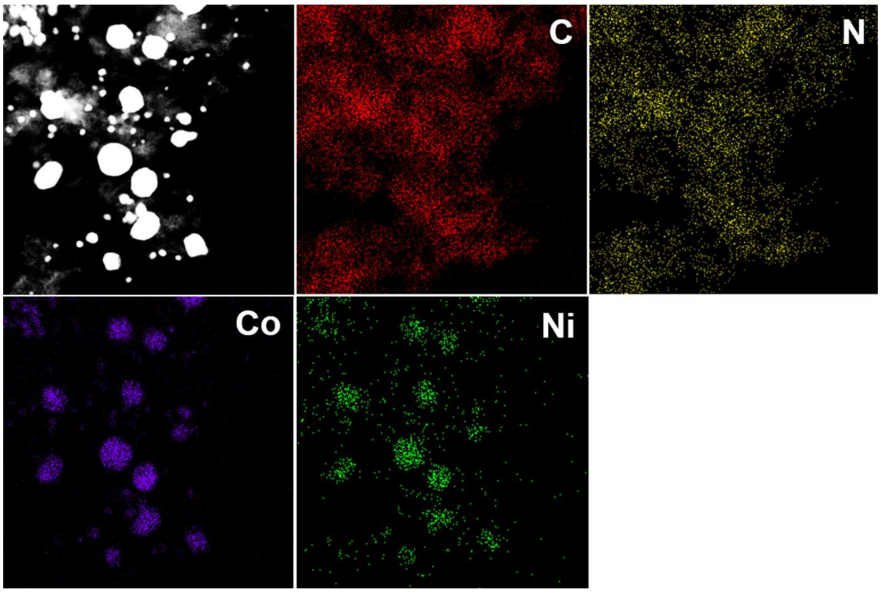

**Fig. S12.** HAADF-STEM and EDS of NC-$Co_{0.49}Ni_{0.51}$.

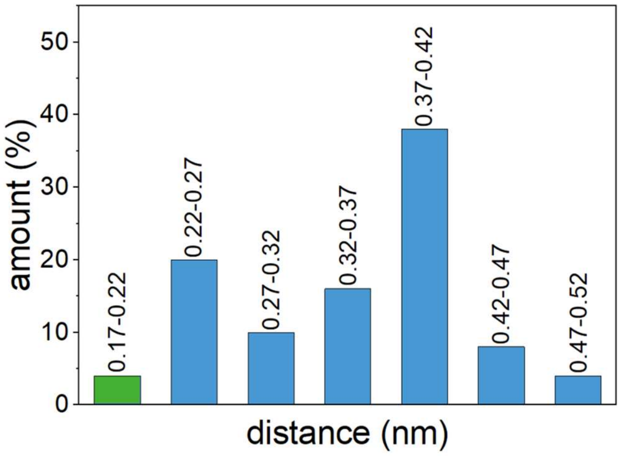


**Fig. S13.** Distribution of distances between adjacent bright dots in Fig. 1f. The distances less than 2.2 Å can be attributed to the overlap of metal atoms at different heights.

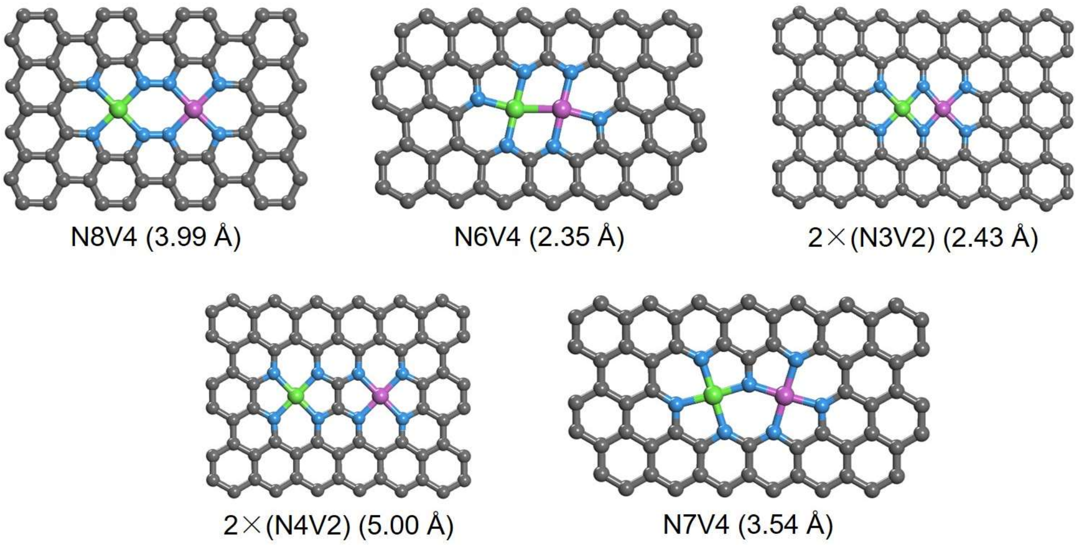


**Fig. S14.** The five different dual metal sites models denoted as N8V4 (3.99 Å), N6V4 (2.35 Å), 2×(N3V2) (2.43 Å), 2×(N4V2) (5.00 Å), and N7V4 (3.54 Å).

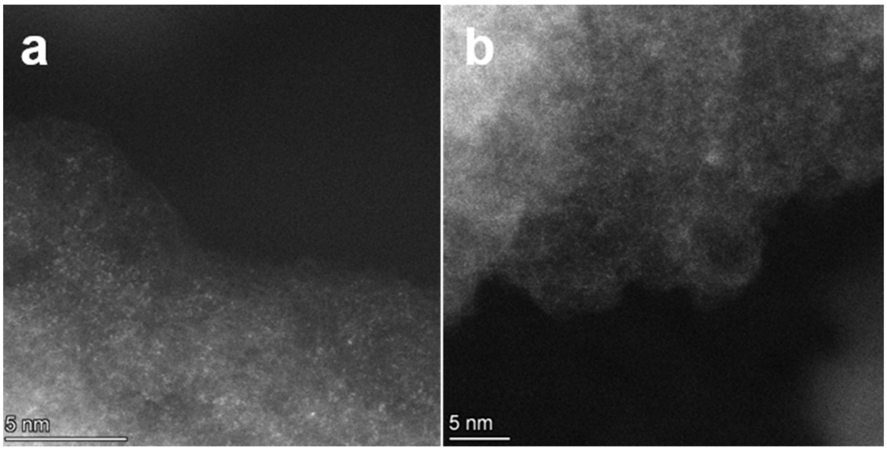


**Fig. S15.** HAADF-STEM images of (a) AD-$Co_1Ni_0$ and (b) AD-$Co_0Ni_1$.

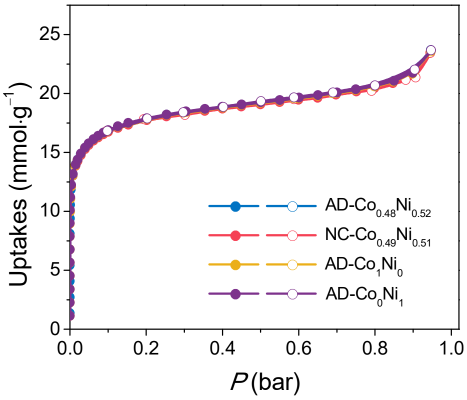


Fig. S16. 77 K $N_2$ adsorption isotherms of AD-$Co_1Ni_0$, AD-$Co_0Ni_1$, AD-$Co_{0.48}Ni_{0.52}$, NC-$Co_{0.49}Ni_{0.51}$.

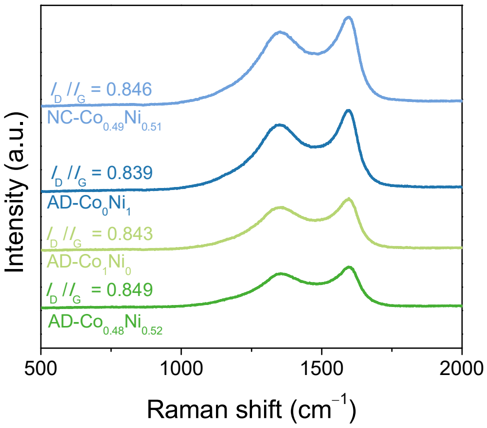


**Fig. S17.** Raman spectra of AD-$Co_{0.48}Ni_{0.52}$, AD-$Co_1Ni_0$, AD-$Co_0Ni_1$, and NC-$Co_{0.49}Ni_{0.51}$.

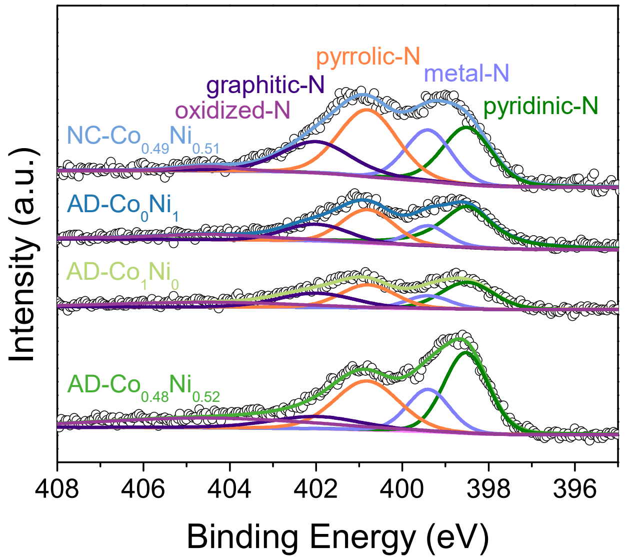


**Fig. S18.** High resolution XPS spectrum of AD-$Co_{0.48}Ni_{0.52}$, AD-$Co_1Ni_0$, AD-$Co_0Ni_1$, and NC-$Co_{0.49}Ni_{0.51}$ at the N 1s region.

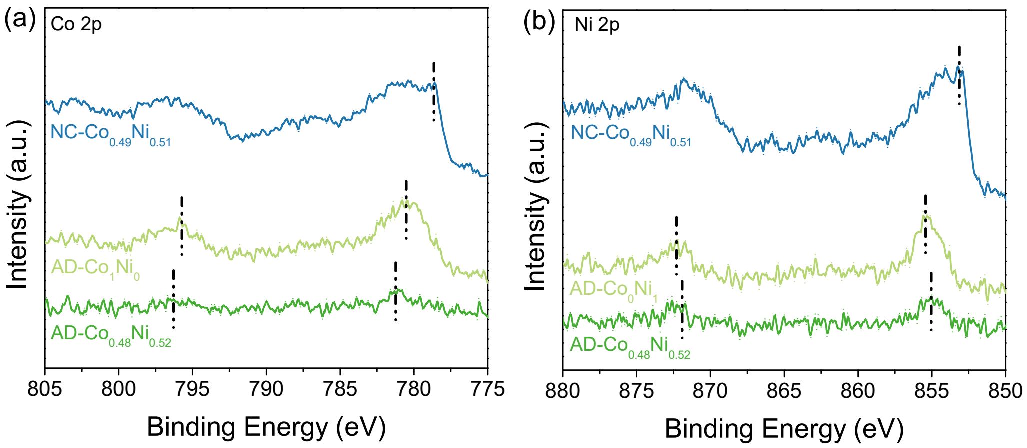


**Fig. S19.** High resolution XPS spectra of AD-$Co_{0.48}Ni_{0.52}$, AD-$Co_1Ni_0$, AD-$Co_0Ni_1$, and NC-$Co_{0.49}Ni_{0.51}$ at the (a) Co 2p and (b) Ni 2p region.

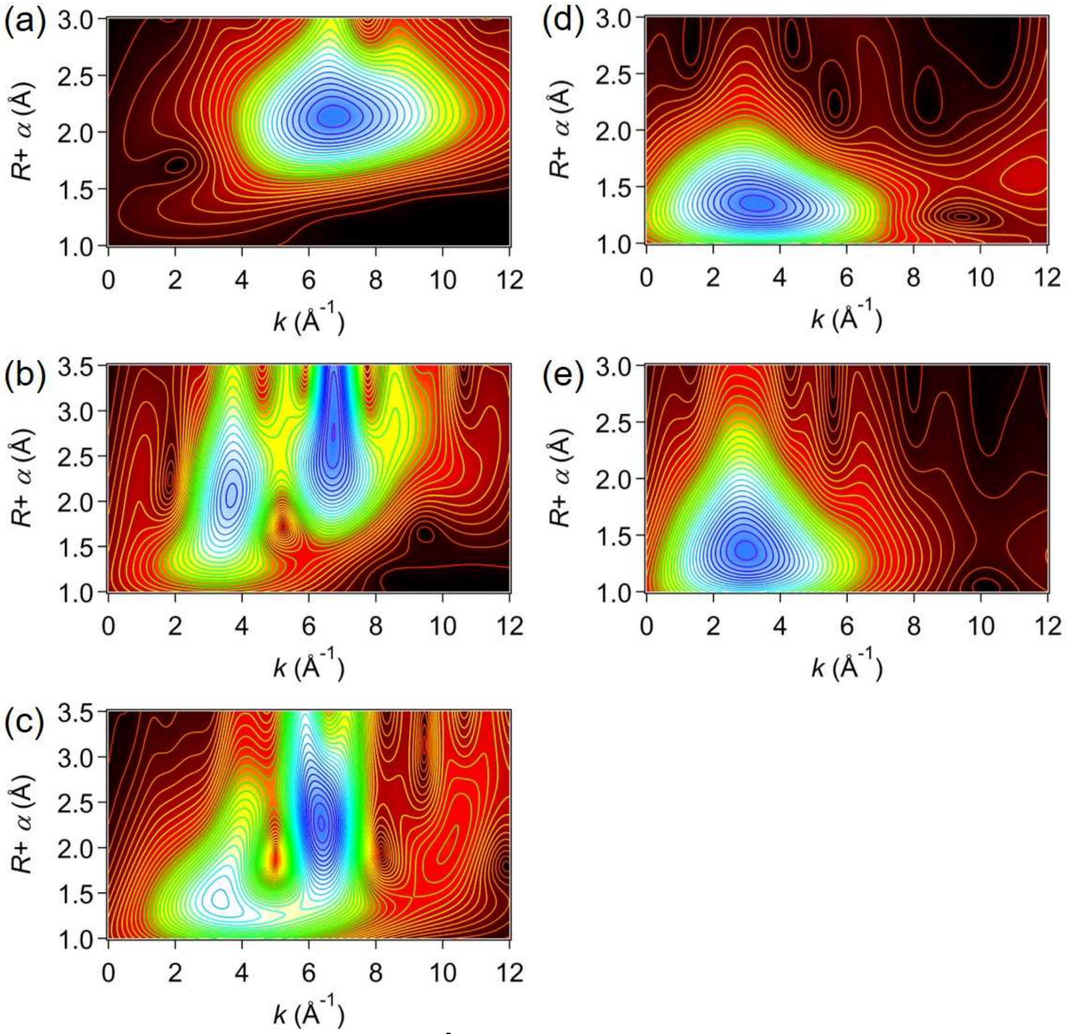


**Fig. S20.** Wavelet transforms for the $k^3$-weighted Co K-edge EXAFS signals of (a) Co foil, (b) CoO, (c) $Co_3O_4$, (d) AD-$Co_{0.48}Ni_{0.52}$, and (e) AD-$Co_1Ni_0$.

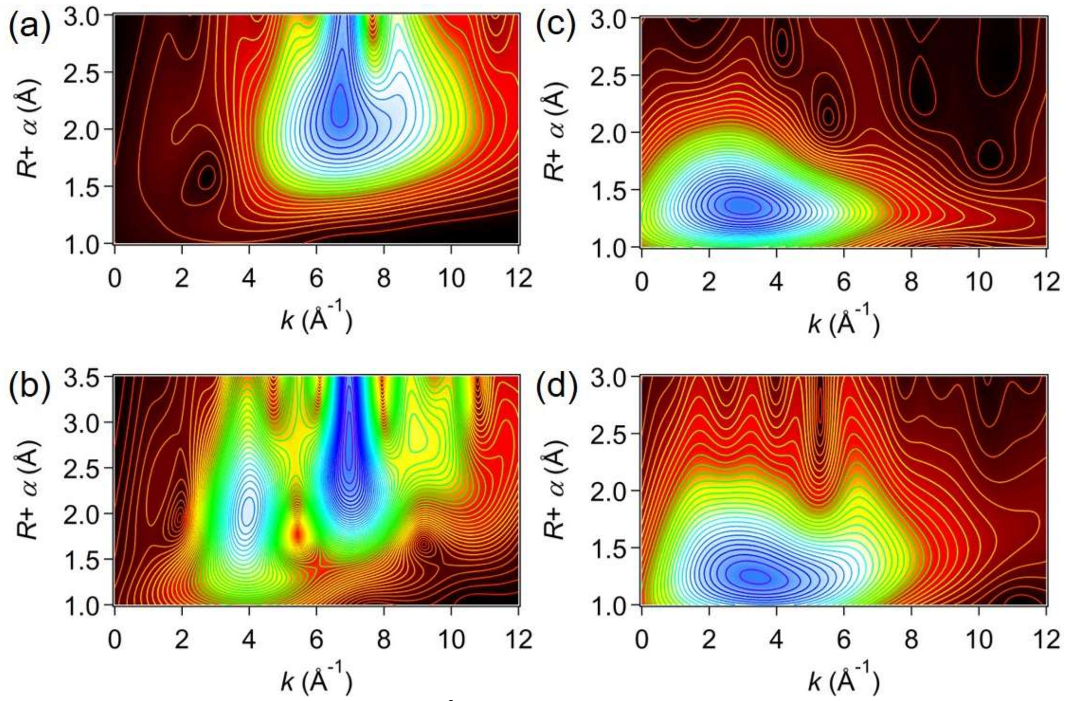


**Fig. S21.** Wavelet transforms for the $k^3$-weighted Ni K-edge EXAFS signals of (a) Ni foil, (b) NiO, (c) AD-$Co_{0.48}Ni_{0.52}$, and (d) AD-$Co_0Ni_1$.

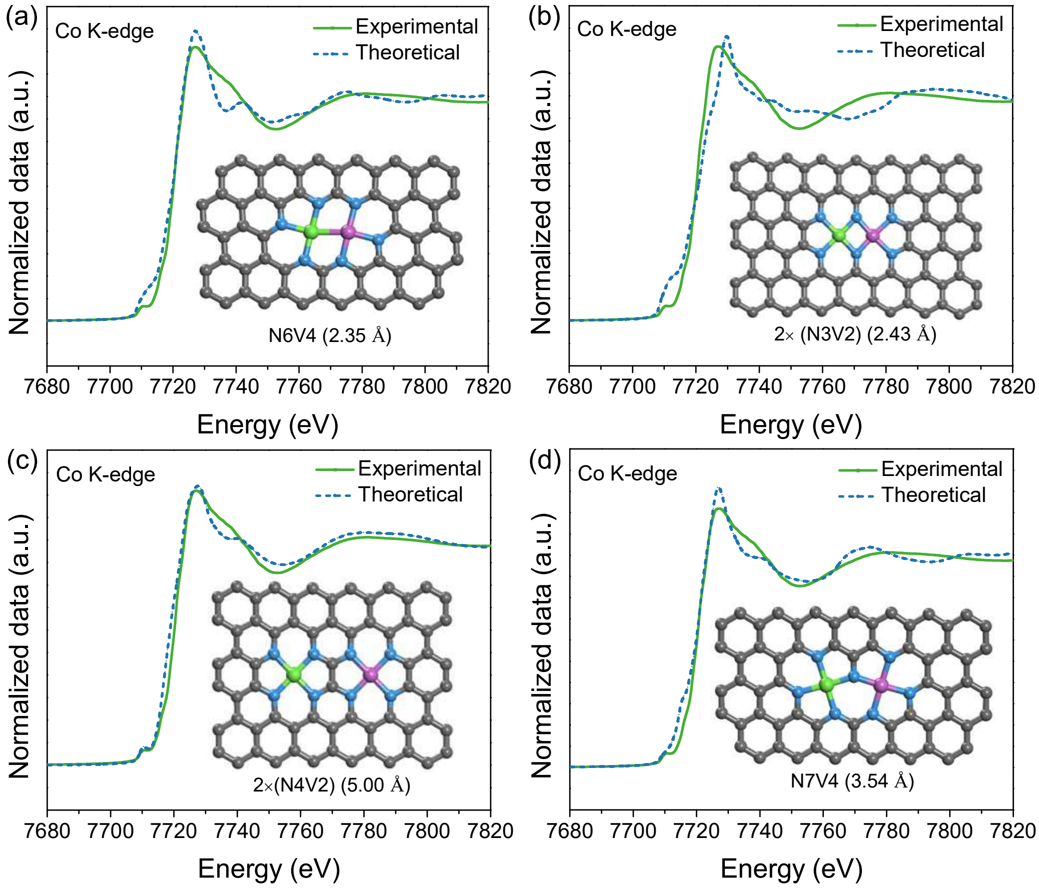


**Fig. S22.** Theoretical Co K-edge XANES spectra of the proposed models of (a) N6V4-CoNi, (b) 2×(N3V2)-CoNi, (c) 2×(N4V2)-CoNi, and (d) N7V4-CoNi.

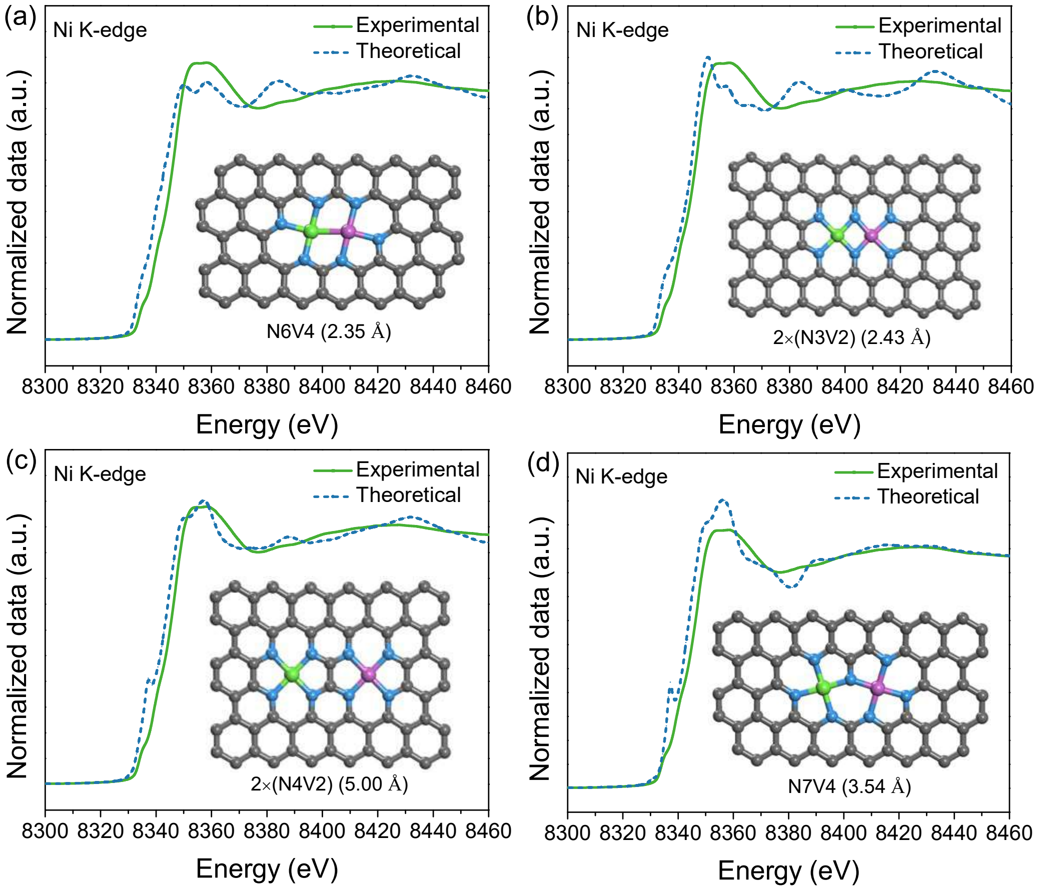


**Fig. S23.** Theoretical Ni K-edge XANES spectra of the proposed models of (a) N6V4-CoNi, (b) 2×(N3V2)-CoNi, (c) 2×(N4V2)-CoNi, and (d) N7V4-CoNi.

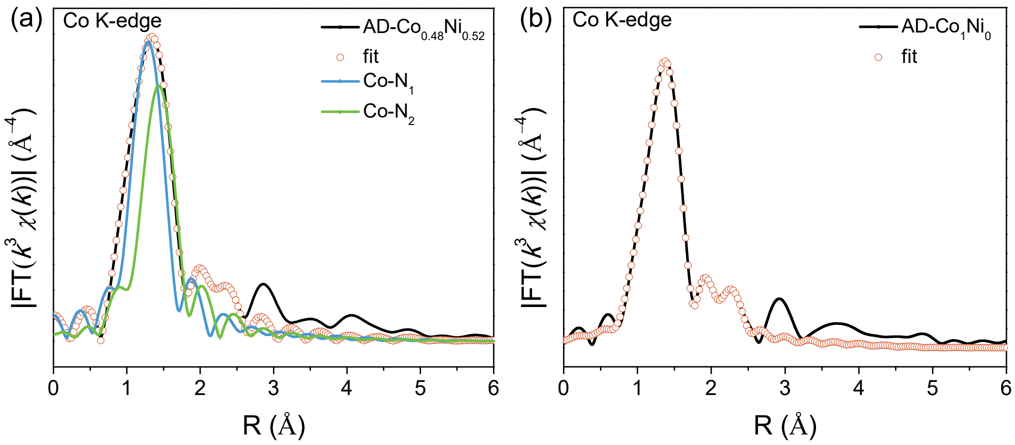


**Fig. S24.** Co K-edge EXAFS spectra fitting of (a) AD-$Co_{0.48}Ni_{0.52}$ and (b) AD-$Co_1Ni_0$.

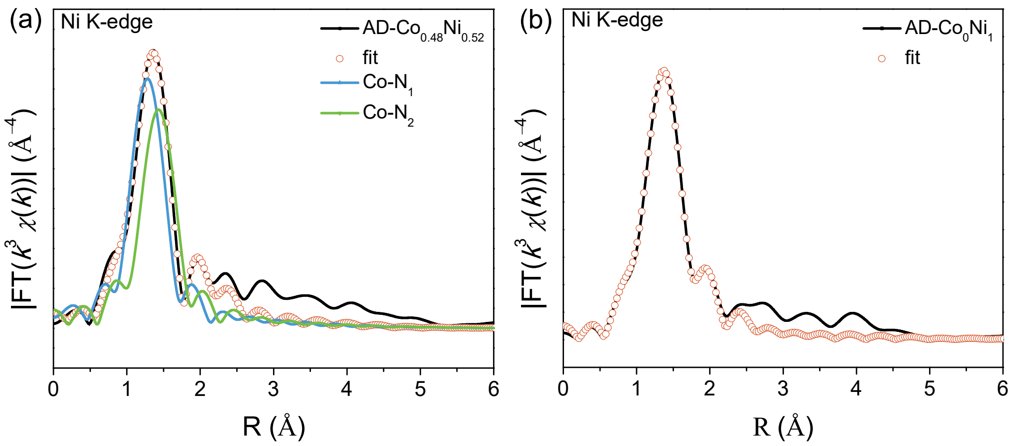


**Fig. S25.** Ni K-edge EXAFS spectra fitting of (a) AD-$Co_{0.48}Ni_{0.52}$ and (b) AD-$Co_0Ni_1$.

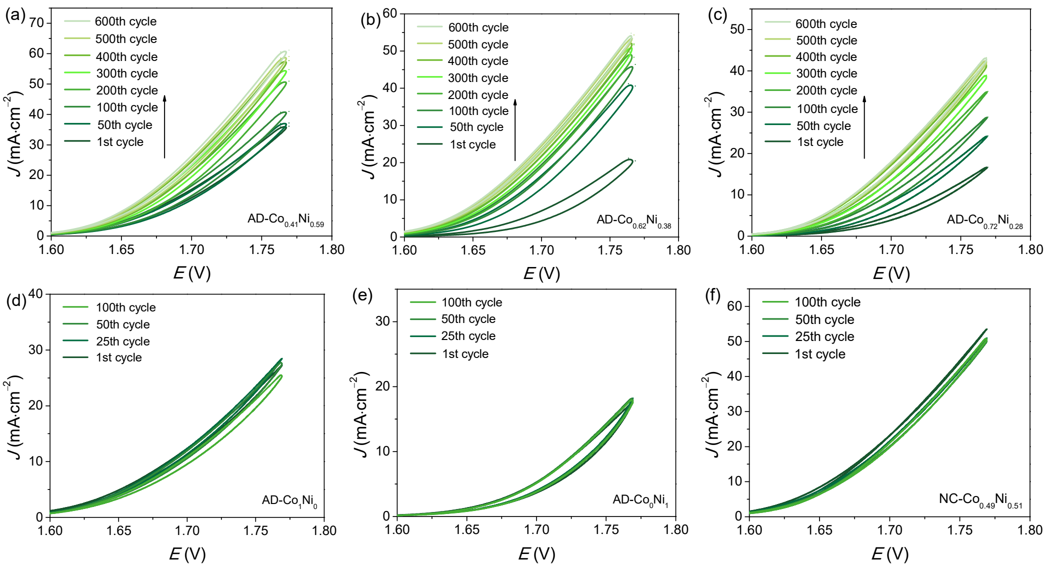


**Fig. S26.** OER CV curves of (a) AD-$Co_{0.41}Ni_{0.59}$, (b) AD-$Co_{0.62}Ni_{0.38}$, (c) AD-$Co_{0.72}Ni_{0.28}$, (d) AD-$Co_1Ni_0$, (e) AD-$Co_0Ni_1$, and (f) NC-$Co_{0.49}Ni_{0.51}$.

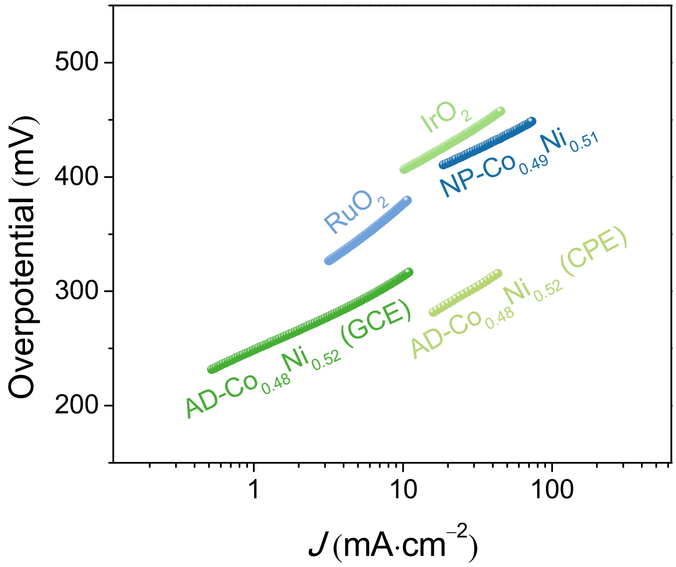


**Fig. S27.** OER Tafel slopes for AD-$Co_{0.48}Ni_{0.52}$ (GCE), AD-$Co_{0.48}Ni_{0.52}$ (CPE), NC-$Co_{0.49}Ni_{0.51}$, $RuO_2$, and $IrO_2$.

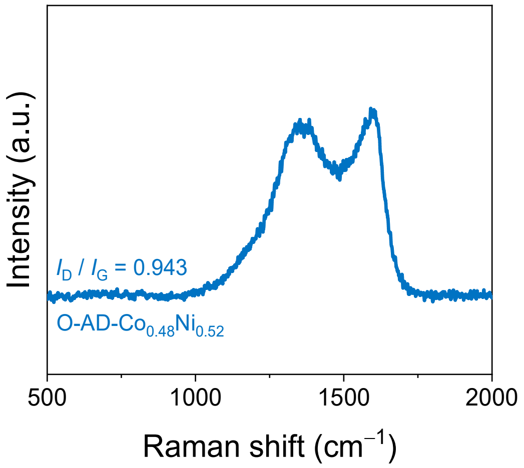


**Fig. S28.** Raman spectra of AD-$Co_{0.48}Ni_{0.52}$ after OER.

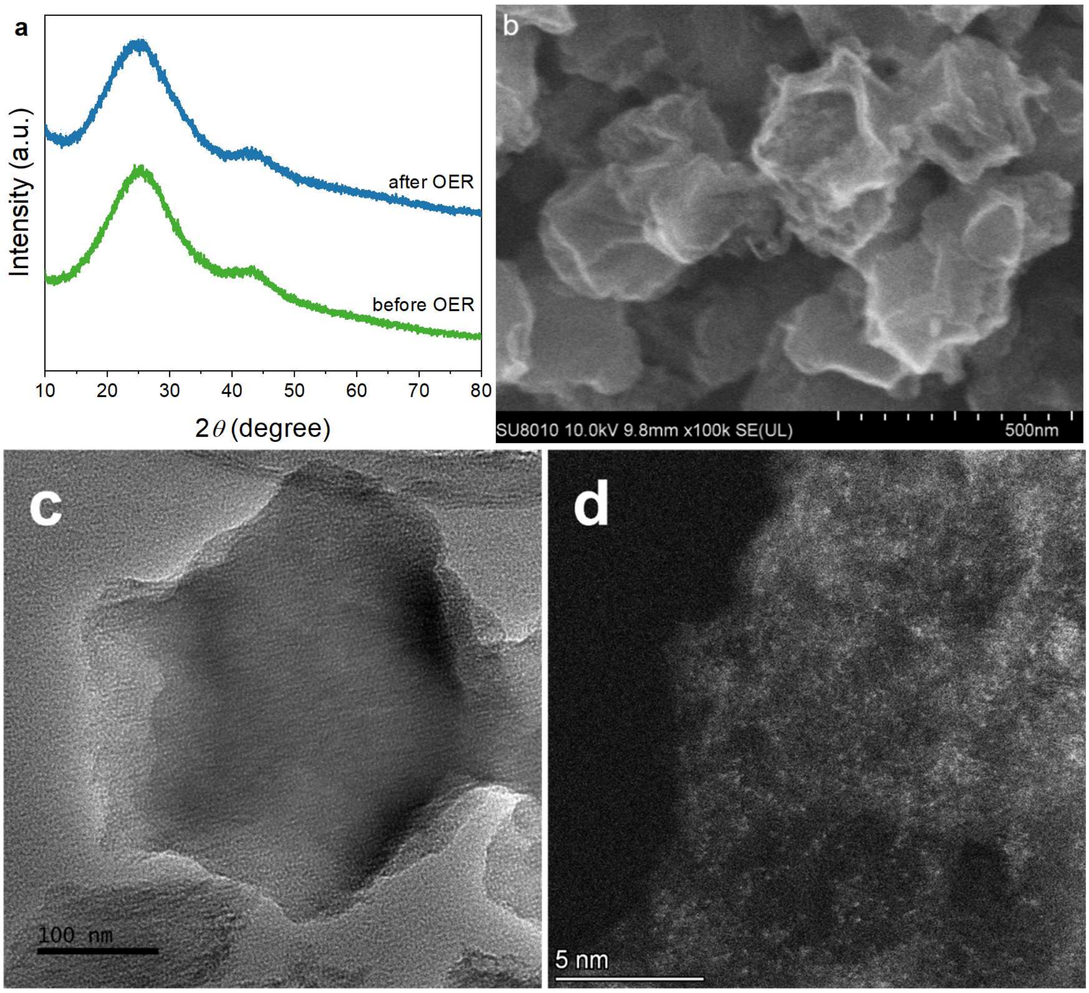


**Fig. S29.** (a) PXRD patterns of AD-$Co_{0.48}Ni_{0.52}$ before and after OER. (b) SEM, (c)TEM and (d) HADDF-STEM image of AD-$Co_{0.48}Ni_{0.52}$ after OER.

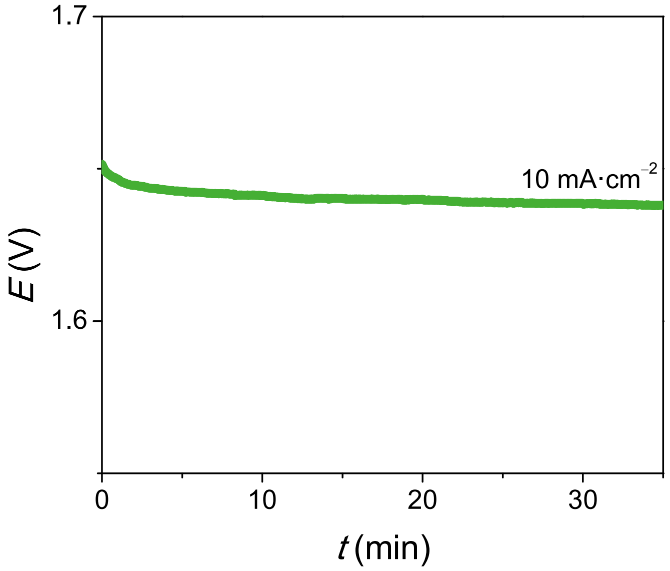


**Fig. S30.** OER chronopotentiometry curve of AD-$Co_{0.48}Ni_{0.52}$ at 10 mA·$cm^{-2}$ during operando ATR-FTIR (Fig. 4a).

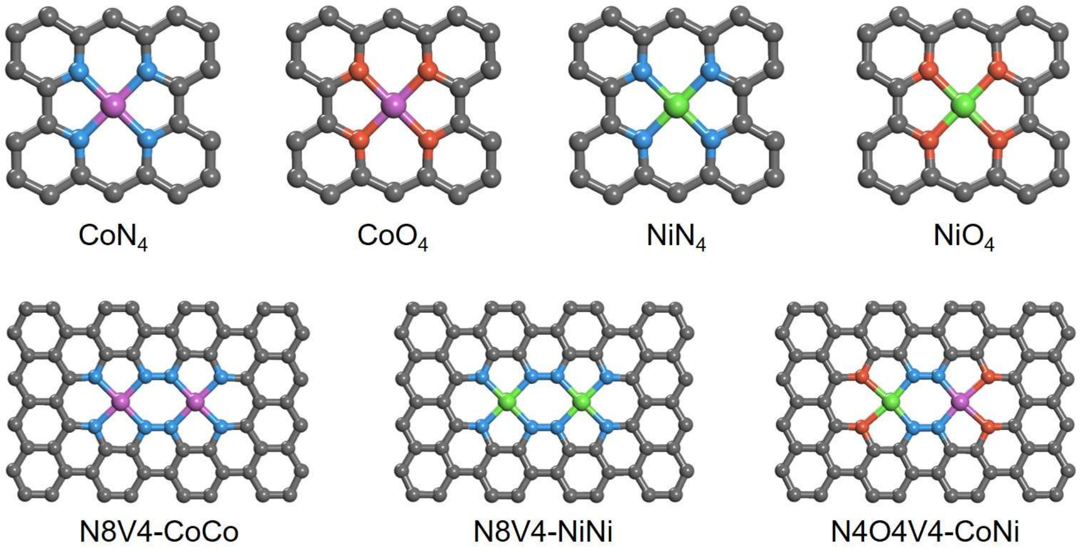


**Fig. S31.** Structures of the $CoN_4$, $CoO_4$, $NiN_4$, $NiO_4$, N8V4-CoCo, N8V4-NiNi, and N4O4V4-CoNi models.

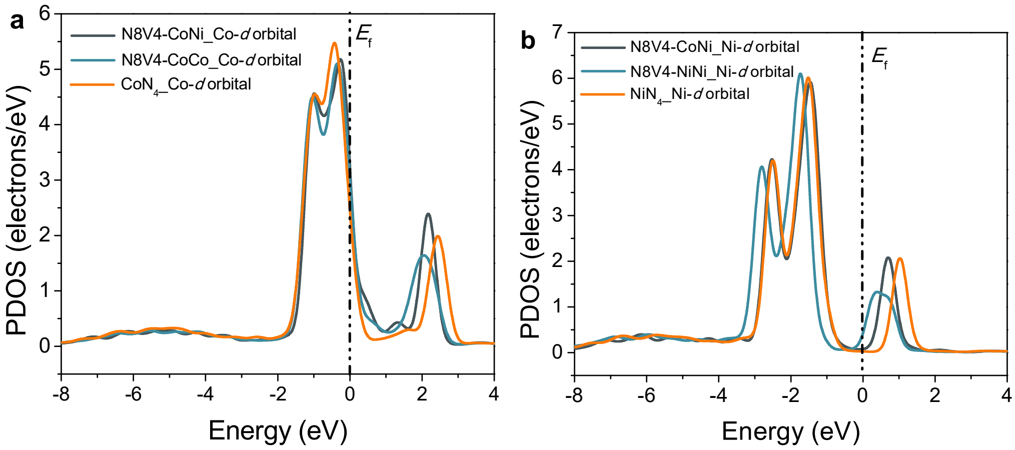


**Fig. S32.** DFT derived partial DOS of (a) Co-d-orbital in N8V4-CoNi, N8V4-CoCo and $CoN_4$ and (b) Ni-d-orbital in N8V4-CoNi, N8V4-NiNi and $NiN_4$.

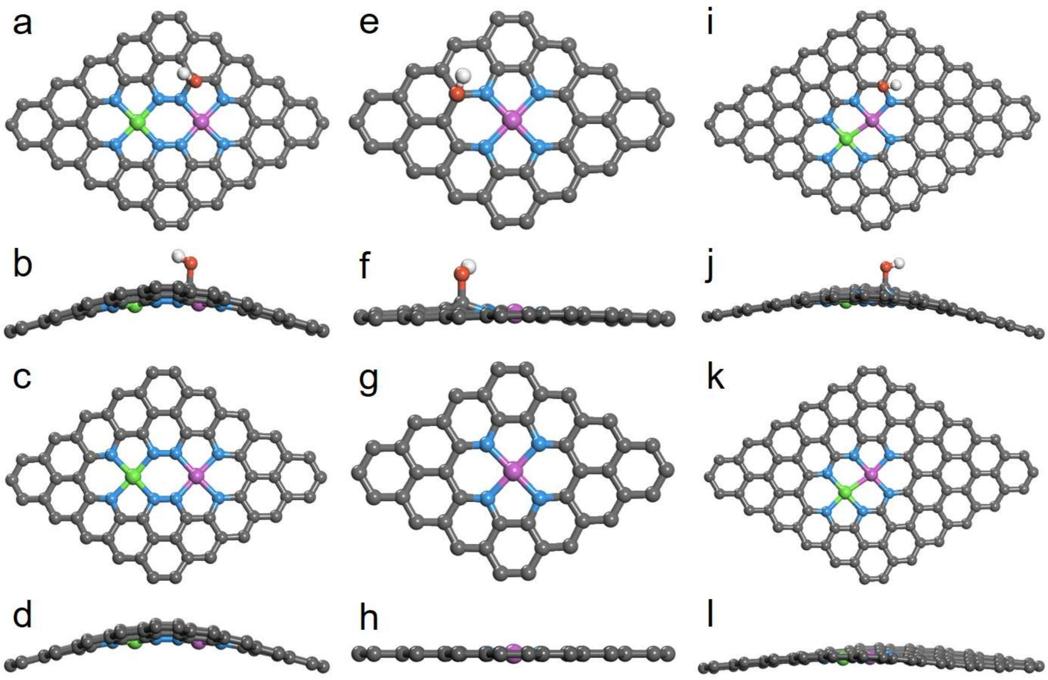


**Fig. S33.** The graphene matrix inlaid with (a and b) N8V4-CoNi-OH, (c and d) N8V4-CoNi, (e and f) $CoN_4$-OH, (g and h) $CoN_4$, (i and j) N6V4-CoNi-OH, and (k and l) N6V4-CoNi.

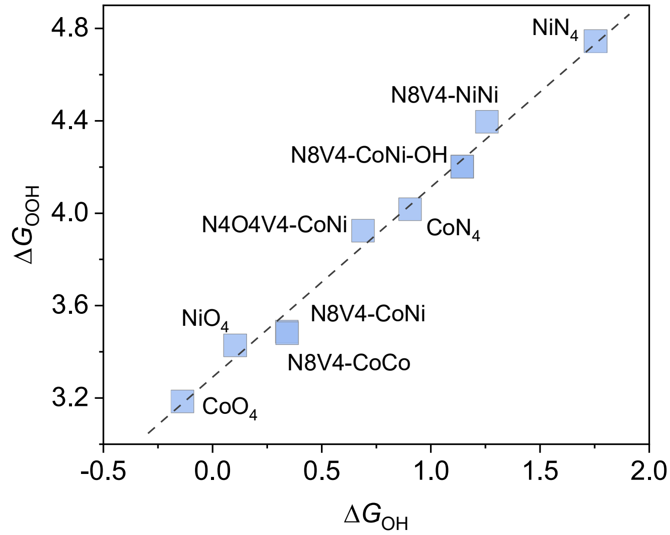


**Fig. S34.** Scaling relation between the binding energies for OH and OOH.

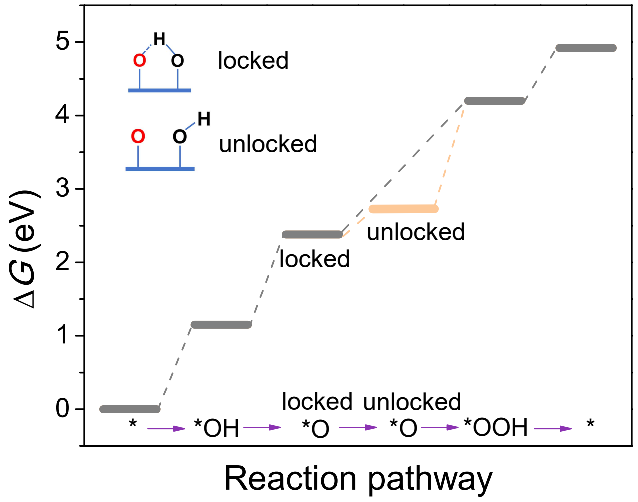


**Fig. S35.** Scaling relationship of AD-$Co_{0.48}Ni_{0.52}$ for OER.

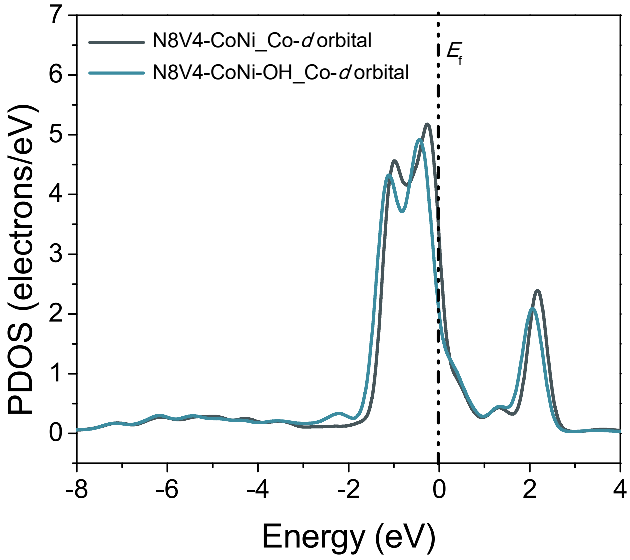


**Fig. S36.** DFT derived partial DOS of (a) Co-d-orbital in N8V4-CoNi, N8V4-CoNi-OH.

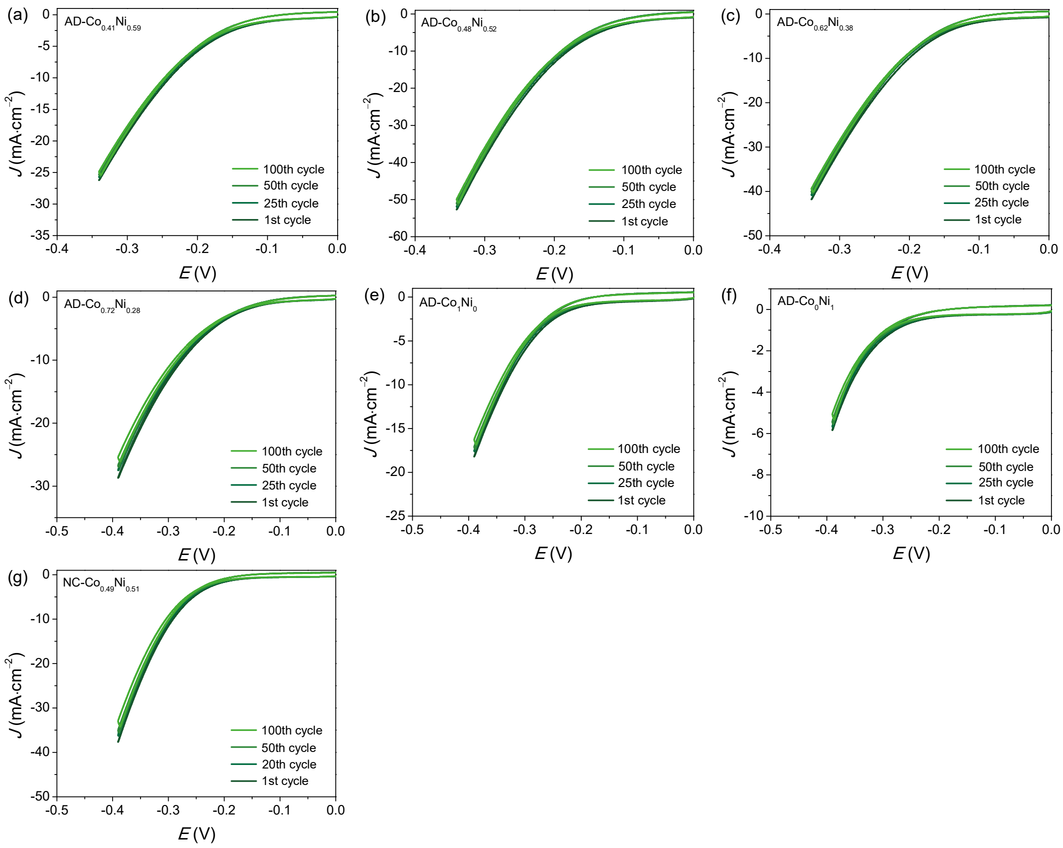


**Fig. S37.** HER CV curves of (a) AD-$Co_{0.41}Ni_{0.59}$, (b) AD-$Co_{0.48}Ni_{0.52}$, (c) AD-$Co_{0.62}Ni_{0.38}$, (d) AD-$Co_{0.72}Ni_{0.28}$, (e) AD-$Co_1Ni_0$, (f) AD-$Co_0Ni_1$, and (g) NC-$Co_{0.49}Ni_{0.51}$.

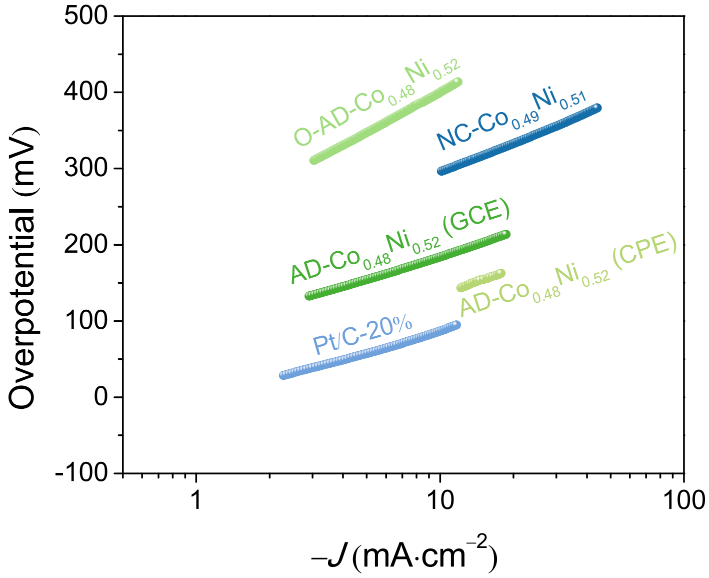


**Fig. S38.** HER Tafel slopes for AD-$Co_{0.48}Ni_{0.52}$ (GCE), AD-$Co_{0.48}Ni_{0.52}$ (CPE), NC-$Co_{0.49}Ni_{0.51}$, O-AD-$Co_{0.48}Ni_{0.52}$, and Pt/C-20%.

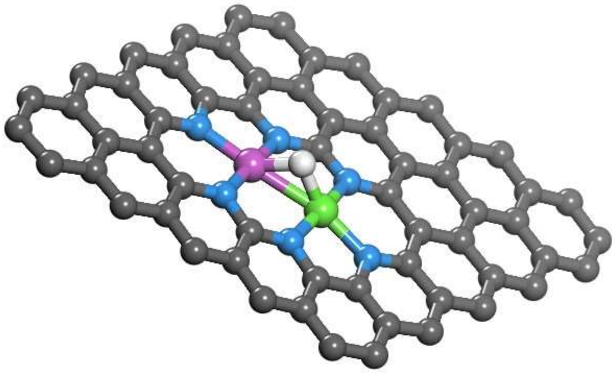

**Fig. S39.** Hydrogen intermediate adsorbed on the N6V4-CoNi model.

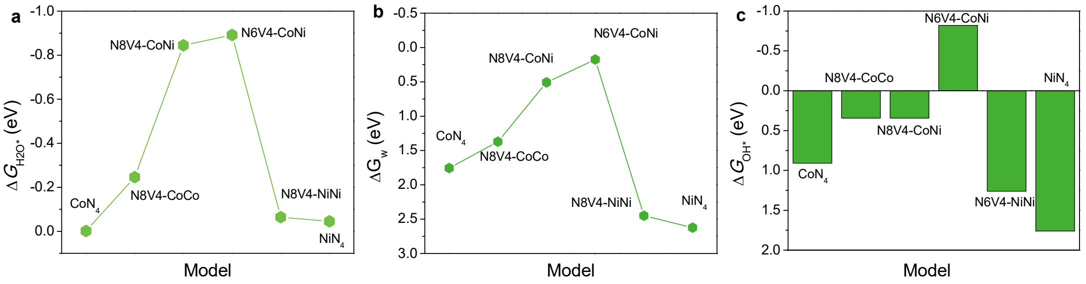


Fig. 40. Scaling relationship of (a) $H_2O$ adsorption energy, (b) $H_2O$ dissociation energy, and (c) OH* adsorption energy in $CoN_4$, N8V4-CoCo, N8V4-CoNi, N6V4-CoNi, N8V4-NiNi and $NiN_4$ for HER.

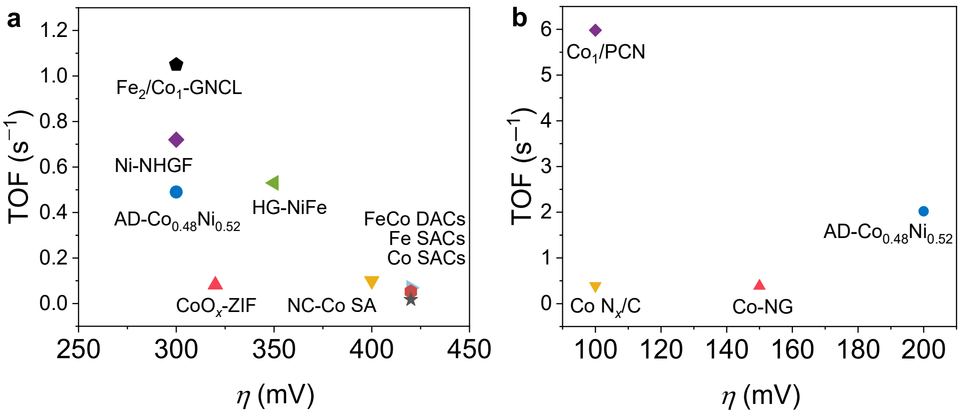


Fig. S41. TOF values of AD-$Co_{0.48}Ni_{0.52}$ and other atomically dispersed electrocatalysts.

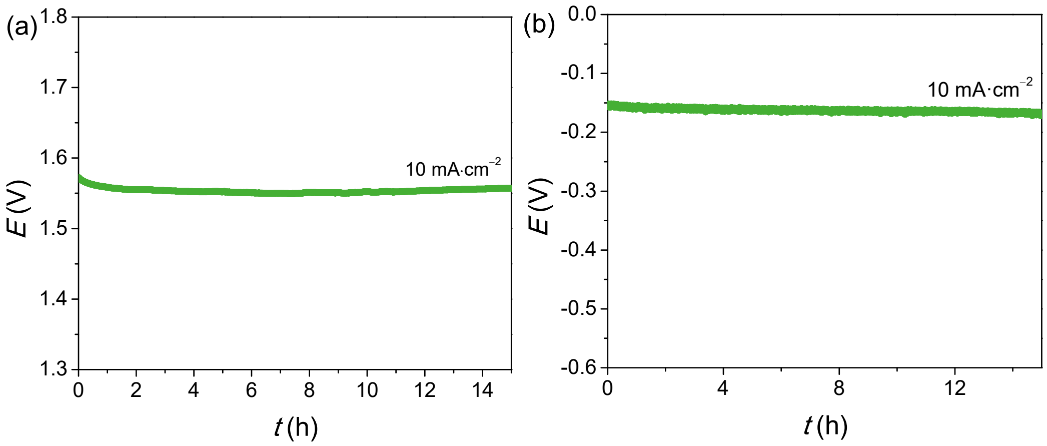

**Fig. 42.** (a) OER and (b) HER chronopotentiometry curves for AD-$Co_{0.48}Ni_{0.52}$ coated on CPE.

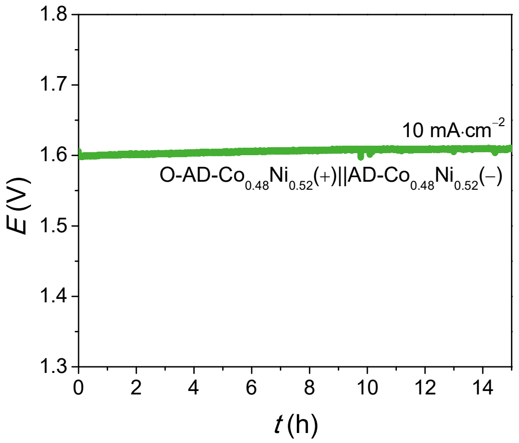

**Fig. S43.** Chronopotentiometry curve of O-AD-$Co_{0.48}Ni_{0.52}$(+)‖AD-$Co_{0.48}Ni_{0.52}$(−) for overall water splitting.

**Table S1.** The feeding parameters for Co/Ni-doped MAF-4.

| Feeding amounts | | | Pyrolysis product |
|---|---|---|---|
| *z* | *c* | *n* | |
| 3.5 | 0.109 | 0 | AD-$Co_1Ni_0$ |
| 3.5 | 0.078 | 0.124 | AD-$Co_{0.72}Ni_{0.28}$ |
| 3.5 | 0.068 | 0.168 | AD-$Co_{0.62}Ni_{0.38}$ |
| 3.5 | 0.055 | 0.238 | AD-$Co_{0.48}Ni_{0.52}$ |
| 3.5 | 0.044 | 0.261 | AD-$Co_{0.41}Ni_{0.59}$ |
| 3.5 | 0 | 0.475 | AD-$Co_0Ni_1$ |
| 3.4 | 0.081 | 0.358 | Not studied because of the uncertain structure of metal species |
| 3.3 | 0.110 | 0.479 | |
| 3.2 | 0.175 | 0.741 | NC-$Co_{0.49}Ni_{0.51}$ |

**Table S2.** Structural parameters extracted from the Co K-edge and Ni K-edge EXAFS fitting. ($S_0^2$ = 0.81 for Co K-edge and 0.85 for Ni K-edge)

| Sample | Edge | Scattering pair | $N$ | $R$ (Å) | $\sigma^2$ ($10^{-3}$ Å$^2$) | $\Delta E_0$ (eV) | $R$ factor |
|---|---|---|---|---|---|---|---|
| AD-$Co_{0.48}Ni_{0.52}$ | Co | Co-N1 | 2.2 | 1.93 | 5.4 | 1.0 | 0.005 |
| | | Co-N2 | 1.9 | 1.97 | 5.9 | | |
| | Ni | Ni-N1 | 2.1 | 1.94 | 5.1 | 1.5 | 0.006 |
| | | Ni-N2 | 2.0 | 1.99 | 5.7 | | |
| AD-$Co_1Ni_0$ | Co | Co-N | 4.1 | 1.95 | 4.6 | 1.5 | 0.007 |
| AD-$Co_0Ni_1$ | Ni | Ni-N | 3.9 | 1.96 | 4.9 | 1.5 | 0.004 |

$S_0^2$ is the amplitude reduction factor; $N$ is the coordination number; $R$ is interatomic distance (the bond length between Co/Ni central atoms and surrounding coordination atoms); $\sigma^2$ is the Debye-Waller factor value (a measure of thermal and static disorder in absorber-scatterer distances); $\Delta E_0$ is edge-energy shift (the difference between the zero kinetic energy value of the sample and that of the theoretical model); $R$ factor is used to value the goodness of the fitting.

Error bounds that characterize the structural parameters obtained by EXAFS spectroscopy were estimated as $N \pm 20\%$; $R \pm 1\%$; $\sigma^2 \pm 20\%$; $\Delta E_0 \pm 20\%$.

**Table S3.** Determination of the F:O atomic ratio of AD-$Co_{0.48}Ni_{0.52}$ before and after OER.

| | F | O | F:O |
|---|---|---|---|
| Before OER | 41.35 | 5.66 | 7.31:1 |
| After OER | 36.78 | 6.96 | 5.28:1 |

Fluorine (F) was from Nafion.

**Table S4.** The energy change after oxidation to form C-OH.

| Model | before oxidation | *E* (eV) | after oxidation | *E* (eV) | $\Delta E$ (eV) |
|---|---|---|---|---|---|
| N8V4-CoNi | 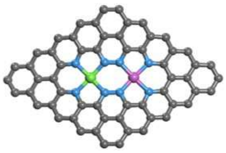 | −14149.6 | 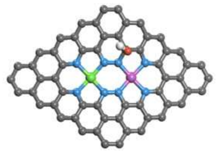 | −14604.6 | 1.08 |
| $CoN_4$ | 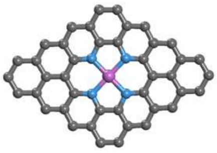 | −12647.9 | 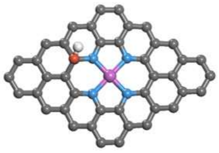 | −13102.9 | 1.63 |
| N6V4-CoNi | 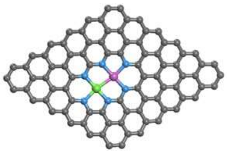 | −20348.6 | 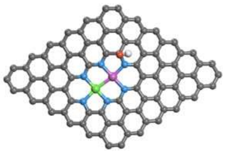 | −20803.9 | 1.48 |

**Table S5.** The calculated energy of the oxidation position on N8V4-CoNi.

| before oxidation | $E$ (eV) | after oxidation | $E$ (eV) | $\Delta E$ (eV) |
|---|---|---|---|---|
| 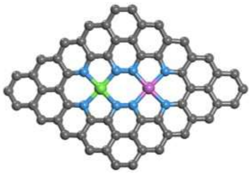 | −14149.6 | 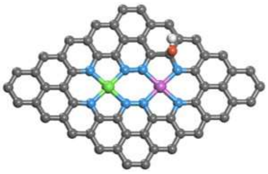 | −14604.2 | 1.512 |
| | | 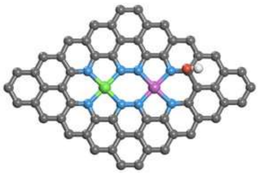 | −14603.8 | 1.860 |
| | | 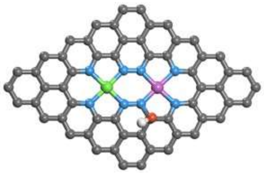 | −14604.0 | 1.658 |
| | | 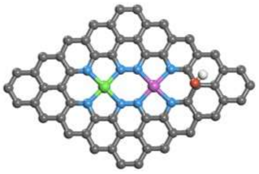 | −14604.0 | 1.692 |
| | | 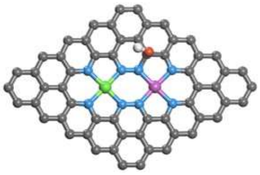 | −14604.6 | 1.080 |

**Table S6.** Benchmark electrocatalytic performances of ADCs.

| Catalysts | Electrolyte | $\eta_{10\text{-OER}}$ / mV | $\eta_{10\text{-HER}}$ / mV | Substrate | Refs. |
|---|---|---|---|---|---|
| AD-$Co_{0.48}Ni_{0.52}$ | 1.0 M KOH | 313 | 183 | GCE | this work |
| | | 264 | 132 | CPE | |
| A-Ni@DG | 1 M KOH | 270 | 150 | GCE | [2] |
| DG | | 340 | N.A. | | |
| Fe-$N_4$ SAs/NPC | 1.0 M KOH | 430 | 202 | GCE | [3] |
| CoSA/N,S-HCS | 1 M KOH | 306 | 165 | CPE | [4] |
| Ru/Co-N-C | 1 M KOH | 276 | 19 | CPE | [5] |
| FeCo-DACs/NC | 1.0 M | 370 | N.A. | CPE | [6] |
| NiFe-CNG | 1 M KOH | 270 | N.A. | GCE | [7] |
| NiFe-DASC | 1 M KOH | 310 | N.A. | GCE | [8] |
| Co-Fe-N-C | 1 M KOH | 360 | N.A. | GCE | [9] |
| | | 321 | N.A. | CC | |
| $Fe_2$/$Co_1$-GNCL | 1 M KOH | 350 | N.A. | GCE | [10] |
| $Fe_2$-GNCL | | 355 | N.A. | | |
| NiFe@g-$C_3N_4$/CNT | 1 M KOH | 326 | N.A. | GCE | [11] |
| FeNi@PCN | 1 M KOH | 310 | N.A. | GCE | [12] |
| CoNi-SAs/NC | 1 M KOH | 340 | N.A. | CC | [13] |
| a-NiCo/NC | 1.0 M KOH | 252 | N.A. | CC | [14] |
| Co-$C_3N_4$/CNT | 1 M KOH | 380 | N.A. | GCE | [15] |
| Co SA@NCF/CNF | 1 M KOH | 400 | N.A. | CC | [16] |
| Ni-O-G SACs | 1 M KOH | 328 | N.A. | CC | [17] |
| 0.7-Co@NG-750 | 1.0 M KOH | 386 | N.A. | GCE | [18] |
| Ni-NHGF | 1 M KOH | 331 | N.A. | GCE | [19] |
| Co-NHGF | | 402 | N.A. | | |
| Fe-NHGF | | 488 | N.A. | | |
| NHGF | | 494 | N.A. | | |
| Mn-NG | 1.0 M KOH | 337 | N.A. | GCE | [20] |
| Mn-G | | 459 | N.A. | | |
| NC-Co SA | 1 M KOH | 360 | N.A. | CC | [21] |
| Ni-O-G SACs | 1 M KOH | 224 | N.A. | CC | [22] |
| Co-NG-5010-10 | 1 M KOH | 470 | N.A. | GCE | [23] |
| Ni-CN-200 | 1.0 M KOH | 310 (onset) | N.A. | GCE | [24] |
| S,N-Fe/N/C-CNT | 0.1 M KOH | 370 | N.A. | GCE | [25] |
| $CoN_4$/NG | 0.1 M KOH | 380 | N.A. | GCE | [26] |
| Ni-$N_4$/GHSs/Fe-$N_4$ | 0.1 M KOH | 390 | N.A. | GCE | [27] |
| (Fe,Co)-SA/CS | 0.1 M KOH | 360 | N.A. | Ni foam | [28] |
| Co-NG | 1 M KOH | N.A. | 270 | GCE | [29] |
| $CoN_x$/G | 1.0 M KOH | N.A. | 170 | GCE | [30] |
| $Co_1$/PCN | 1.0 M KOH | N.A. | 89 | Ni foam | [31] |
| $Co_1$/CN | | N.A. | 138 | | |

**Table S7.** Benchmark performances of bifunctional electrocatalysts for overall water splitting in 1 M KOH. (Blue: ADCs, green: quasi-bifunctional electrocatalysts, red: other bifunctional electrocatalysts)

| Catalysts | Substrate | Cell voltages-$\eta_{10}$ / V | Refs. |
|---|---|---|---|
| O-AD-$Co_{0.48}Ni_{0.52}$(+)‖AD-$Co_{0.48}Ni_{0.52}$(−) | CPE | 1.60 | this work |
| Fe-$N_4$ SAs/NPC | CPE | 1.67 | [3] |
| CoSA/N,S-HCS | CPE | 1.64 | [4] |
| Ru/Co-N-C | CPE | 1.50 | [5] |
| Fe-$O_2$cat(+)‖Fe-$H_2$cat(−) | Fe foam | 1.65 | [32] |
| R-$CoO_x$@CN(+)‖R-$CoO_x$@CN(−) | Ni foam | 1.6 | [33] |
| $Ni_2P/NiO_x$ (+)‖$Ni_2P$(−) | Ni foam | 1.63 | [34] |
| $Ir_1$@Co/NC | CPE | 1.60 | [35] |
| CoP/NCNHP | CPE | 1.64 | [36] |
| CoP/rGO | CPE | 1.7 | [37] |
| Co-S sheets | CPE | 1.743 | [38] |
| $Co_3O_4$ NCs | CPE | 1.91 | [39] |
| Co-P/NC | GCE | 1.71 | [40] |
| $Co_1Mn_1$CH | Ni foam | 1.68 | [41] |
| NiFe LDH | Ni foam | 1.7 | [42] |
| $NiCo_2O_4$ | Ni foam | 1.65 | [43] |
| $Co_{0.85}$Se/NiFe-LDH | graphite foil | 1.67 | [44] |
| $Ni_3S_2$ | Ni foam | 1.76 | [45] |
| Cu@$CoS_x$ | Cu foam | 1.5 | [46] |
| Cu@NiFe LDH | Cu foam | 1.54 | [47] |

**References**


1. B. Ravel, M. Newville, ATHENA, ARTEMIS, HEPHAESTUS: data analysis for X-ray absorption spectroscopy using IFEFFIT. J. Synchrotron Radiat. **12**, 537-541 (2005). https://doi.org/10.1107/S0909049505012719
2. L. Zhang, Y. Jia, G. Gao, X. Yan, N. Chen, et al., Graphene defects trap atomic Ni species for hydrogen and oxygen evolution reactions. Chem **4**, 285-297 (2018). https://doi.org/10.1016/j.chempr.2017.12.005
3. Y. Pan, S. Liu, K. Sun, X. Chen, B. Wang, et al., A bimetallic Zn/Fe polyphthalocyanine-derived single-atom Fe-$N_4$ catalytic site: a superior trifunctional catalyst for overall water splitting and Zn-air batteries. Angew. Chem. Int. Ed. **57**, 8614-8618 (2018). https://doi.org/10.1002/anie.201804349
4. Z. Zhang, X. Zhao, S. Xi, L. Zhang, Z. Chen, et al., Atomically dispersed cobalt trifunctional electrocatalysts with tailored coordination environment for flexible rechargeable Zn-air battery and self-driven water splitting. Adv. Energy Mater. **10**, 2002896 (2020). https://doi.org/10.1002/aenm.202002896
5. C. Rong, X. Shen, Y. Wang, L. Thomsen, T. Zhao, et al., Electronic structure engineering of single-atom Ru sites via Co-$N_4$ sites for bifunctional pH-universal water splitting. Adv. Mater. **34**, 2110103 (2022). https://doi.org/10.1002/adma.202110103
6. M. Liu, N. Li, S. Cao, X. Wang, X. Lu, et al., A "pre-constrained metal twins" strategy to prepare efficient dual-metal-atom catalysts for cooperative oxygen electrocatalysis. Adv. Mater. **34**, 2107421 (2022). https://doi.org/10.1002/adma.202107421
7. W. Wan, Y. Zhao, S. Wei, C.A. Triana, J. Li, et al., Mechanistic insight into the active centers of single/dual-atom Ni/Fe-based oxygen electrocatalysts. Nat. Commun. **12**, 5589 (2021). https://doi.org/10.1038/s41467-021-25811-0
8. Z. Zeng, L.Y. Gan, H. Bin Yang, X. Su, J. Gao, et al., Orbital coupling of hetero-diatomic nickel-iron site for bifunctional electrocatalysis of $CO_2$ reduction and oxygen evolution. Nat. Commun. **12**, 4088 (2021). https://doi.org/10.1038/s41467-021-24052-5
9. L. Bai, C.S. Hsu, D.T.L. Alexander, H.M. Chen, X. Hu, A cobalt-iron double-atom catalyst for the oxygen evolution reaction. J. Am. Chem. Soc. **141**, 14190-14199 (2019). https://doi.org/10.1021/jacs.9b05268
10. Y.-S. Wei, L. Sun, M. Wang, J. Hong, L. Zou, et al., Fabricating dual-atom iron catalysts for efficient oxygen evolution reaction: a heteroatom modulator approach. Angew. Chem. Int. Ed. **59**, 16013-16022 (2020). https://doi.org/10.1002/anie.202007221
11. D. Liu, S. Ding, C. Wu, W. Gan, C. Wang, et al., Synergistic effect of an atomically dual-metal doped catalyst for highly efficient oxygen evolution. J. Mater. Chem. A **6**, 6840-6846 (2018). https://doi.org/10.1039/c8ta00550h
12. C. Wu, X. Zhang, Z. Xia, M. Shu, H. Li, et al., Insight into the role of Ni-Fe dual sites in the oxygen evolution reaction based on atomically metal-doped polymeric carbon nitride. J. Mater. Chem. A **7**, 14001-14010 (2019). https://doi.org/10.1039/c9ta03163d
13. X. Han, X. Ling, D. Yu, D. Xie, L. Li, et al., Atomically dispersed binary Co-Ni sites in nitrogen-doped hollow carbon nanocubes for reversible oxygen reduction and evolution. Adv. Mater. **31**, 1905622 (2019). https://doi.org/10.1002/adma.201905622
14. Z. Pei, X.F. Lu, H. Zhang, Y. Li, D. Luan, et al., Highly efficient electrocatalytic oxygen evolution over atomically dispersed synergistic Ni/Co dual sites. Angew. Chem. Int. Ed. **61**, 202207537 (2022) https://doi.org/10.1002/anie.202207537.
15. Y. Zheng, Y. Jiao, Y. Zhu, Q. Cai, A. Vasileff, et al., Molecule-level g-$C_3N_4$ coordinated transition metals as a new class of electrocatalysts for oxygen electrode reactions. J. Am. Chem. Soc. **139**, 3336-3339 (2017). https://doi.org/10.1021/jacs.6b13100
16. D. Ji, L. Fan, L. Li, S. Peng, D. Yu, et al., Atomically transition metals on self-supported porous carbon flake arrays as binder-free air cathode for wearable zinc-air batteries. Adv. Mater. **31**, 1808267 (2019). https://doi.org/10.1002/adma.201808267
17. Y. Xu, W. Zhang, Y. Li, P. Lu, Z.-S. Wu, A general bimetal-ion adsorption strategy to prepare nickel single atom catalysts anchored on graphene for efficient oxygen evolution reaction. J. Energy Chem. **43**, 52-57 (2020). https://doi.org/10.1016/j.jechem.2019.08.006
18. Q. Zhang, Z. Duan, M. Li, J. Guan, Atomic cobalt catalysts for the oxygen evolution reaction. Chem. Commun. **56**, 794-797 (2020). https://doi.org/10.1039/c9cc09007j
19. H. Fei, J. Dong, Y. Feng, C.S. Allen, C. Wan, et al., General synthesis and definitive structural identification of $MN_4C4$ single-atom catalysts with tunable electrocatalytic activities. Nat. Catal. **1**, 63-72 (2018). https://doi.org/10.1038/s41929-017-0008-y
20. J. Guan, Z. Duan, F. Zhang, S.D. Kelly, R. Si, et al., Water oxidation on a mononuclear manganese

heterogeneous catalyst. Nat. Catal. **1**, 870-877 (2018). https://doi.org/10.1038/s41929-018-0158-6
21. W. Zang, A. Sumboja, Y. Ma, H. Zhang, Y. Wu, et al., Single Co atoms anchored in porous N-doped carbon for efficient zinc-air battery cathodes. ACS Catal. **8**, 8961-8969 (2018). https://doi.org/10.1021/acscatal.8b02556
22. Y. Li, Z.S. Wu, P. Lu, X. Wang, W. Liu, et al., High-valence nickel single-atom catalysts coordinated to oxygen sites for extraordinarily activating oxygen evolution reaction. Adv. Sci. **7**, 1903089 (2020). https://doi.org/10.1002/advs.201903089
23. Y. Zhang, W. Li, L. Lu, W. Song, C. Wang, et al., Tuning active sites on cobalt/nitrogen doped graphene for electrocatalytic hydrogen and oxygen evolution. Electrochim. Acta. **265**, 497-506 (2018). https://doi.org/10.1016/j.electacta.2018.01.203
24. S. Ohn, S.Y. Kim, S.K. Mun, J. Oh, Y.J. Sa, et al., Molecularly dispersed nickel-containing species on the carbon nitride network as electrocatalysts for the oxygen evolution reaction. Carbon **124**, 180-187 (2017). https://doi.org/10.1016/j.carbon.2017.08.039
25. P. Chen, T. Zhou, L. Xing, K. Xu, Y. Tong, et al., Atomically dispersed iron-nitrogen species as electrocatalysts for bifunctional oxygen evolution and reduction reactions. Angew. Chem. Int. Ed. **56**, 610-614 (2017). https://doi.org/10.1002/anie.201610119
26. L. Yang, L. Shi, D. Wang, Y. Lv, D. Cao, Single-atom cobalt electrocatalysts for foldable solid-state Zn-air battery. Nano Energy **50**, 691-698 (2018). https://doi.org/10.1016/j.nanoen.2018.06.023
27. J. Chen, H. Li, C. Fan, Q. Meng, Y. Tang, et al., Dual single-atomic Ni-$N_4$ and Fe-$N_4$ sites constructing Janus hollow graphene for welective oxygen electrocatalysis. Adv. Mater. **32**, 2003134 (2020). https://doi.org/10.1002/adma.202003134
28. V. Jose, H. Hu, E. Edison, W. Manalastas, Jr., H. Ren, et al., Modulation of single atomic Co and Fe sites on hollow carbon nanospheres as oxygen electrodes for rechargeable Zn-air batteries. Small Methods **5**, 2000751 (2021). https://doi.org/10.1002/smtd.202000751
29. H. Fei, J. Dong, M.J. Arellano-Jimenez, G. Ye, N. Dong Kim, et al., Atomic cobalt on nitrogen-doped graphene for hydrogen generation. Nat. Commun. **6**, 8668 (2015). https://doi.org/10.1038/ncomms9668
30. H.W. Liang, S. Bruller, R. Dong, J. Zhang, X. Feng, et al., Molecular metal-$N_x$ centres in porous carbon for electrocatalytic hydrogen evolution. Nat. Commun. **6**, 7992 (2015). https://doi.org/10.1038/ncomms8992
31. L. Cao, Q. Luo, W. Liu, Y. Lin, X. Liu, et al., Identification of single-atom active sites in carbon-based cobalt catalysts during electrocatalytic hydrogen evolution. Nat. Catal. **2**, 134-141 (2018). https://doi.org/10.1038/s41929-018-0203-5
32. X. Zou, Y. Wu, Y. Liu, D. Liu, W. Li, et al., In situ generation of bifunctional, efficient Fe-based catalysts from mackinawite iron sulfide for water splitting. Chem **4**, 1139-1152 (2018). https://doi.org/10.1016/j.chempr.2018.02.023
33. H. Jin, J. Wang, D. Su, Z. Wei, Z. Pang, et al., In situ cobalt-cobalt oxide/N-doped carbon hybrids as superior bifunctional electrocatalysts for hydrogen and oxygen evolution. J. Am. Chem. Soc. **137**, 2688-2694 (2015). https://doi.org/10.1021/ja5127165
34. L.-A. Stern, L. Feng, F. Song, X. Hu, $Ni_2P$ as a Janus catalyst for water splitting: the oxygen evolution activity of Ni2P nanoparticles. Energy Environ. Sci. **8**, 2347-2351 (2015). https://doi.org/10.1039/c5ee01155h
35. W.H. Lai, L.F. Zhang, W.B. Hua, S. Indris, Z.C. Yan, et al., General π-electron-assisted strategy for Ir, Pt, Ru, Pd, Fe, Ni single-atom electrocatalysts with bifunctional active sites for highly efficient water splitting. Angew. Chem. Int. Ed. **58**, 11868-11873 (2019). https://doi.org/10.1002/anie.201904614
36. Y. Pan, K. Sun, S. Liu, X. Cao, K. Wu, et al., Core-shell ZIF-8@ZIF-67-derived CoP nanoparticle-embedded N-doped carbon nanotube hollow polyhedron for efficient overall water splitting. J. Am. Chem. Soc. **140**, 2610-2618 (2018). https://doi.org/10.1021/jacs.7b12420
37. L. Jiao, Y.X. Zhou, H.L. Jiang, Metal-organic framework-based CoP/reduced graphene oxide: high-performance bifunctional electrocatalyst for overall water splitting. Chem. Sci. **7**, 1690-1695 (2016). https://doi.org/10.1039/c5sc04425a
38. J. Wang, H.X. Zhong, Z.L. Wang, F.L. Meng, X.B. Zhang, Integrated three-dimensional carbon paper/carbon tubes/cobalt-sulfide sheets as an efficient electrode for overall water splitting. ACS Nano **10**, 2342-2348 (2016). https://doi.org/10.1021/acsnano.5b07126
39. S. Du, Z. Ren, J. Zhang, J. Wu, W. Xi, et al., $Co_3O_4$ nanocrystal ink printed on carbon fiber paper as a large-area electrode for electrochemical water splitting. Chem. Commun. **51**, 8066-8069 (2015). https://doi.org/10.1039/c5cc01080b

40. B. You, N. Jiang, M. Sheng, S. Gul, J. Yano, et al., High-performance overall water splitting electrocatalysts derived from cobalt-based metal-organic frameworks. Chem. Mater. **27**, 7636-7642 (2015). https://doi.org/10.1021/acs.chemmater.5b02877
41. T. Tang, W.J. Jiang, S. Niu, N. Liu, H. Luo, et al., Electronic and morphological dual modulation of cobalt carbonate hydroxides by Mn doping toward highly efficient and stable bifunctional electrocatalysts for overall water splitting. J. Am. Chem. Soc. **139**, 8320-8328 (2017). https://doi.org/10.1021/jacs.7b03507
42. J. Luo, J.-H. Im, M.T. Mayer, M. Schreier, M.K. Nazeeruddin, et al., Water photolysis at 12.3% efficiency via perovskite photovoltaics and earth-abundant catalysts. Science **345**, 1593-1596 (2014). https://doi.org/10.1126/science.1258307
43. X. Gao, H. Zhang, Q. Li, X. Yu, Z. Hong, et al., Hierarchical $NiCo_2O_4$ hollow microcuboids as bifunctional electrocatalysts for overall water-splitting. Angew. Chem. Int. Ed. **55**, 6290-6294 (2016). https://doi.org/10.1002/anie.201600525
44. Y. Hou, M.R. Lohe, J. Zhang, S. Liu, X. Zhuang, et al., Vertically oriented cobalt selenide/NiFe layered-double-hydroxide nanosheets supported on exfoliated graphene foil: an efficient 3D electrode for overall water splitting. Energy Environ. Sci. **9**, 478-483 (2016). https://doi.org/10.1039/c5ee03440j
45. L.L. Feng, G. Yu, Y. Wu, G.D. Li, H. Li, et al., High-index faceted $Ni_3S_2$ nanosheet arrays as highly active and ultrastable electrocatalysts for water splitting. J. Am. Chem. Soc. **137**, 14023-14026 (2015). https://doi.org/10.1021/jacs.5b08186
46. Y. Liu, Q. Li, R. Si, G.D. Li, W. Li, et al., Coupling sub-nanometric copper clusters with quasi-amorphous cobalt sulfide yields efficient and robust electrocatalysts for water splitting reaction. Adv. Mater. **29**, 1606200 (2017). https://doi.org/10.1002/adma.201606200
47. L. Yu, H. Zhou, J. Sun, F. Qin, F. Yu, et al., Cu nanowires shelled with NiFe layered double hydroxide nanosheets as bifunctional electrocatalysts for overall water splitting. Energy Environ. Sci. **10**, 1820-1827 (2017). https://doi.org/10.1039/c7ee01571b
